# Gut microbiota dysbiosis and aromatic amino acid metabolism alterations: a multi-omics analysis of cognitive impairment following aneurysmal subarachnoid hemorrhage

**DOI:** 10.3389/fmicb.2026.1870309

**Published:** 2026-08-18

**Authors:** Wentian Zhang, Miaomiao Chen, Tingyu Guo, Guopeng Kuang, Ning Ma

**Affiliations:** 1 The First Clinical School of Medicine, Shanxi Medical University, Taiyuan, China; 2 Department of Neurosurgery, Cardiovascular Hospital Affiliated to Shanxi Medical University, Taiyuan, China; 3 Shanxi Key Laboratory of Heart Failure Precision Medicine, Taiyuan, China; 4 Department of Neurosurgery, The First Hospital of Shanxi Medical University, Taiyuan, China; 5 Academy of Medical Sciences, Shanxi Medical University, Taiyuan, China; 6 School of Public Health, Shanxi Medical University, Taiyuan, China

**Keywords:** aneurysmal subarachnoid hemorrhage, cognitive impairment, gut microbiota, metabolites, microbiota-gut-brain axis, multi-omics

## Abstract

**Background:**

Aneurysmal subarachnoid hemorrhage (aSAH) is frequently followed by persistent cognitive impairment, characterized by a complex and multifactorial pathological mechanism. While the role of the “microbiota-gut-brain axis” in neurocognition has garnered increasing attention, the specific ways in which gut microbiota and their derived metabolites might be associated with the development and progression of post-aSAH cognitive impairment remain largely undefined. Consequently, there remains a lack of systematic multi-omics evidence to elucidate these potential underlying associations.

**Methods:**

In this prospective observational study, we enrolled 48 patients with intracranial aneurysms. Among them, patients with aSAH (*n* = 33) were divided into a cognitive impairment group (aSAH-CI, *n* = 18) and a group without cognitive impairment (aSAH-WCI, *n* = 15) based on a 6-month longitudinal neurocognitive assessment. Patients with unruptured intracranial aneurysms (UIA, *n* = 15) served as the control group. We integrated a multi-omics approach encompassing fecal metagenomics, untargeted metabolomics, and serological profiles of inflammation, oxidative stress, and apoptosis to explore the potential correlations between the host and the microbiome, as well as to identify early diagnostic biomarkers.

**Results:**

Fecal metagenomics revealed distinct gut dysbiosis in aSAH-CI patients, characterized by reduced alpha diversity, depletion of beneficial commensals (e.g., *Agathobacter*), and expansion of opportunistic pathogens (e.g., *Enterococcus*). Functional and metabolomic analyses identified a significant alteration in aromatic amino acid biosynthesis. Specifically, tyrosine metabolism was altered, marked by reduced levels of neurotransmitter precursors and elevated neurotoxic trace amines (tyramine and phenylethylamine). Serologically, aSAH-CI patients exhibited heightened systemic inflammation, oxidative stress, and apoptosis. Integrated multi-omics network analysis underscored a strong correlation between elevated trace amines, depleted *Agathobacter*, and systemic pathological indices. Notably, *Agathobacter rectalis* and tyramine demonstrated robust potential as early diagnostic biomarkers for cognitive impairment following aSAH.

**Conclusion:**

Our findings suggest a potential dual-hit correlative signature via the microbiota-gut-brain axis in cognitive impairment following aSAH. We hypothesize that the depletion of aromatic amino acid-producing microbiota correlates with reduced neurotransmitter precursors, theoretically impairing synaptic repair. Concurrently, observed associations among opportunistic pathogens, trace amines, and systemic inflammatory and oxidative stress markers suggest a synergistic effect potentially linked to further neuronal damage.

## 1 Introduction

Subarachnoid hemorrhage (SAH) is a severe cerebrovascular disease. The spontaneous rupture of intracranial aneurysms constitutes the predominant etiology, accounting for approximately 80%–85% of cases (Maher et al., 2020). aSAH is associated with high morbidity and mortality, thereby imposing a significant burden on healthcare systems. A meta-analysis encompassing 75 studies across 32 countries indicated a global annual incidence of spontaneous SAH of 8 per 100,000 individuals, demonstrating an overall declining trend (Etminan et al., 2019). Moreover, recent advancements in microsurgical and endovascular techniques have progressively reduced the mortality and morbidity rates associated with aSAH. Nevertheless, even after surviving the acute phase, 27%–44% of patients with aSAH experience cognitive impairment (Wong et al., 2016). A longitudinal study detailing the long-term trajectory of social cognition and behavioral issues following aSAH demonstrated that these deficits are persistent and exhibit limited potential for recovery (Jorna et al., 2025).

Although the clinical consequences of cognitive impairment following aSAH are widely recognized, its underlying pathophysiological mechanisms remain complex and require further elucidation. Concurrent with advancing research on the microbiota-gut-brain axis, the role of gut microbiota in neurological disorders has garnered increasing attention. Accumulating evidence demonstrates that gut microbiota dysbiosis is closely associated with various cognitive disorders, including schizophrenia spectrum disorders, Alzheimer’s disease, and vascular dementia, suggesting that specific microbial taxa may serve as potential biomarkers for cognitive dysfunction (Frileux et al., 2024; Liu et al., 2025; Warren et al., 2026). The gut microbiota can establish a bidirectional communication system between the gut and the brain through various signaling pathways, including neural, endocrine, and immune pathways, thereby facilitating reciprocal interaction and regulation (Agirman and Hsiao, 2021). Beyond presenting as a severe localized intracranial event, aSAH rapidly precipitates a sympathetic surge and a systemic stress response syndrome (Al-Mufti et al., 2019). This profound pathophysiological cascade can alter gastrointestinal motility and secretory functions, subsequently correlating with shifts in the composition of the gut microbiota (Singh et al., 2016; Morais et al., 2020). Previous studies have demonstrated that the gut microbiota following aSAH is characterized by decreased diversity, enrichment of pro-inflammatory taxa, and depletion of beneficial microbes, such as butyrate-producing bacteria. These microbial shifts may contribute to a pathophysiological foundation for the subsequent development of cognitive impairment by potentially reducing the availability of neuroprotective metabolites (e.g., alpha-linolenic acid and butyrate), facilitating the systemic translocation of pro-inflammatory agents (e.g., lipopolysaccharide), and perturbing amino acid metabolism (Kawabata et al., 2022; Sun et al., 2024; Xu et al., 2024; Csecsei et al., 2025).

Notably, extensive literature has established that gut microbiota dysbiosis is closely associated with the pathological progression of multiple neurological disorders, including stroke, Parkinson’s disease, and Alzheimer’s disease (Benakis et al., 2020). During the progression of these conditions, an aberrant activation of the gut-brain inflammatory axis is frequently observed. The disease-associated neuroinflammation and primary brain injury could potentially be linked to disrupted gut microbial homeostasis (Cryan et al., 2019). In turn, the resultant dysbiotic microbiota and their derived metabolites are thought to modulate and potentially contribute to the inflammatory response within the central nervous system (Singh et al., 2016; Cryan et al., 2019). Furthermore, a compromised intestinal mucosal barrier may facilitate the translocation of pathogenic microbes and their toxic metabolites into the peripheral circulation (Kelly et al., 2015). This systemic dissemination is frequently associated with widespread inflammatory responses and heightened oxidative stress, pathological processes that are recognized as critical contributors to the pathogenesis of cognitive impairment (Heneka et al., 2025; Liu et al., 2026).

To date, direct evidence demonstrating an association between cognitive impairment following aSAH and the gut microbiota is lacking. In the context of aSAH, contemporary research has predominantly focused on the influence of the gut microbiota on the formation and rupture of intracranial aneurysms. Consequently, systematic investigations characterizing the cross-omics associations between gut microbiota dysbiosis and the longitudinal development of cognitive impairment in this patient population remain scarce. To address this critical gap, the present study integrates metagenomic sequencing, untargeted metabolomics, and comprehensive serological profiling. By conducting correlation analyses among differentially abundant microbial taxa, altered metabolites, and serum biomarkers, alongside the construction of association networks and receiver operating characteristic (ROC) curves, we comprehensively delineate a cross-system, multi-omics association network characterizing cognitive impairment following aSAH. Ultimately, this study provides comprehensive multi-omics evidence highlighting acute-phase dysbiosis of the microbiota-gut-brain axis as a potential predictive signature for the longitudinal progression of cognitive impairment following aSAH. Furthermore, our findings offer a novel translational perspective for the development of non-invasive early predictive biomarkers and the exploration of neuroprotective strategies targeting the gut microbiota.

## 2 Materials and methods

### 2.1 Participant recruitment

In this prospective observational study conducted between October 2024 and February 2025 at the Department of Neurosurgery, First Hospital of Shanxi Medical University, we recruited 33 patients with aSAH and 15 concurrent patients with UIA to serve as the control group. To minimize the potential disruption of the gut microbiota associated with craniotomy and postoperative prophylactic antibiotic administration, the cohort was strictly limited to patients undergoing endovascular interventions. Patients were eligible for inclusion if they had a confirmed diagnosis of an intracranial aneurysm and were between 18 and 65 years of age. Primary exclusion criteria encompassed the systemic use of antibiotics or probiotics within the preceding three months, any clinical antibiotic exposure from admission to fecal sample collection, a prior diagnosis of neurodegenerative disease, or severe systemic organ failure. Detailed inclusion and exclusion criteria are outlined in the Supplementary Method 1.

### 2.2 Cognitive evaluation

Cognitive function assessments were independently conducted by two trained psychiatrists who were blinded to the patients’ clinical assignments, neuroimaging characteristics, and gut microbiota profiles. The Montreal Cognitive Assessment (MoCA) and Mini-Mental State Examination (MMSE) were uniformly administered at discharge, and at 3 and 6 months post-discharge. The diagnostic cut-off was established as follows: a total MoCA score of <26 was defined as cognitive impairment. To rigorously adjust for potential bias introduced by educational attainment, one point was added to the total MoCA score for patients with 12 years of education or less. Concurrently, an MMSE score of <27 served as a complementary diagnostic criterion for cognitive impairment (Nasreddine et al., 2005; Luis et al., 2009). Furthermore, a decline of greater than two points in cognitive scores at either the 3- or 6-month post-discharge follow-up intervals was also classified as indicating cognitive impairment. Based on these criteria, the 33 patients with aSAH were classified into two groups: aSAH-CI (*n* = 18) and aSAH-WCI (*n* = 15). In the final outcome assessment of this study, the MoCA score served as the primary sensitive index for screening postoperative cognitive impairment following aSAH, while the MMSE scores served as a supplementary validation for severe cognitive impairment. In the event of a significant discrepancy between the two evaluators (defined as a difference of >2 points on a single scale), a third senior expert was consulted to conduct a re-evaluation and establish consensus.

### 2.3 Clinical data collection and sample collection

**FIGURE 1:**
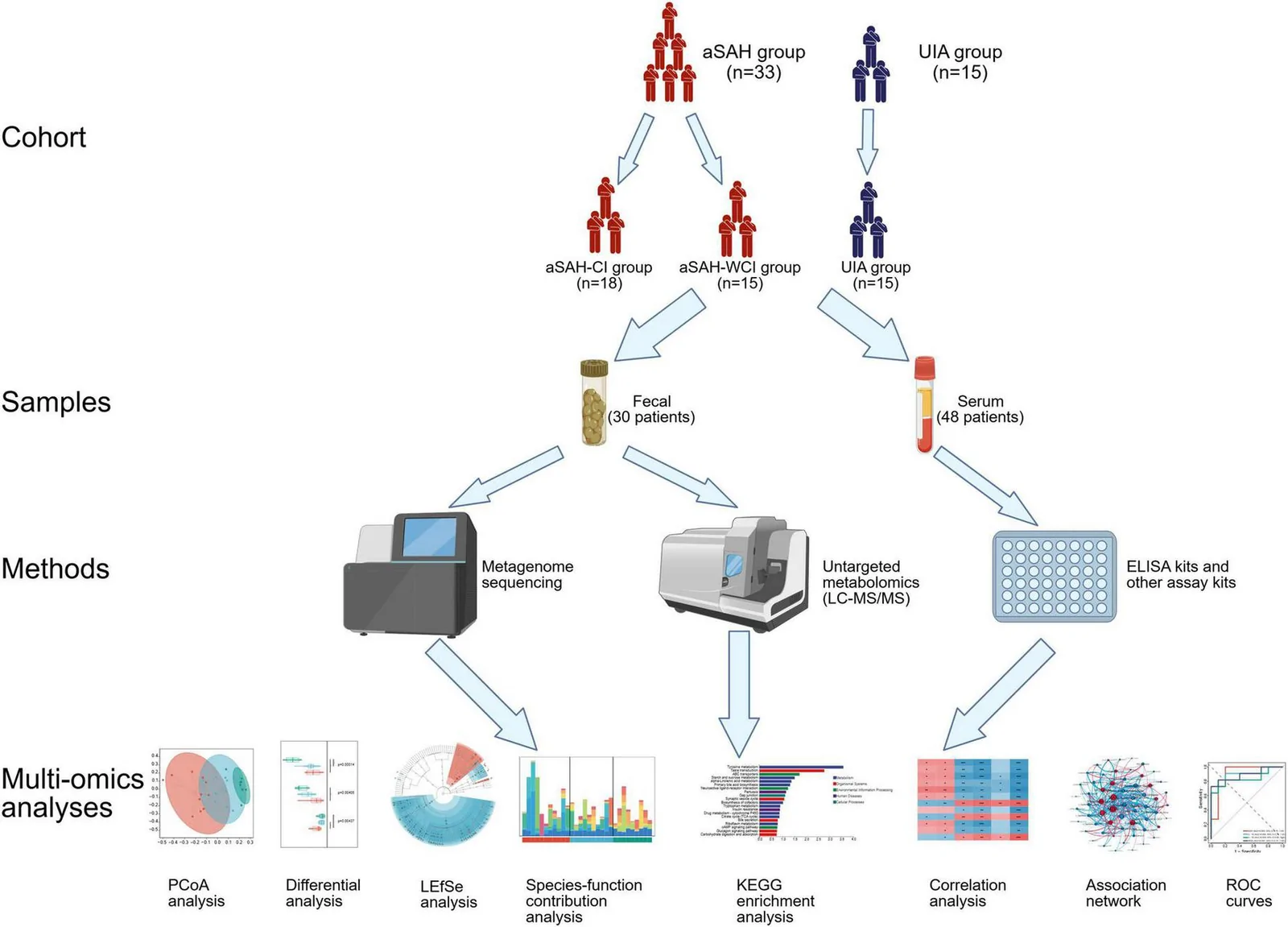
The flowchart of this study. Metagenome sequencing and untargeted metabolomics analysis were performed on fecal samples from 30 patients and serum factor detection was performed on serum samples from 48 patients. Created with BioGDP.com (Jiang et al., 2025).

Clinical data of all participants were obtained upon admission, encompassing demographic characteristics including sex, age, body mass index (BMI), educational attainment, tobacco use, and alcohol consumption; comorbidities including hypertension, diabetes mellitus, and dyslipidemia; aSAH severity including Hunt-Hess grade, Fisher grade, and complications; aneurysm characteristics including location, maximum diameter, height-width ratio, bifurcation status, and multiplicity; and medications including proton pump inhibitors (PPIs), opioids, and laxatives. Fasting venous serum and fecal samples were collected from patients within 24–48 h of admission. Serum samples from the entire cohort were analyzed to quantify specific inflammatory cytokines, including interferon-gamma (IFN-γ), interleukin-1 beta (IL-1β), interleukin-6 (IL-6), interleukin-10 (IL-10), interleukin-18 (IL-18), and tumor necrosis factor-alpha (TNF-α); apoptotic markers, including Bcl-2-associated X protein (BAX), Caspase-3, and cytochrome c (Cyt-C); and oxidative stress indices, including malondialdehyde (MDA), superoxide dismutase (SOD), and reduced glutathione (GSH). Fecal samples were successfully collected from only 30 of the 48 enrolled patients. The remaining 18 patients were unable to defecate within the strict 48-h sampling window due to acute neurocritical constraints, including severe stress, prolonged bed rest, and autonomic dysfunction causing severe constipation or paralytic ileus. Consequently, fecal samples obtained from a subset of 30 patients, comprising 11 with aSAH-CI, 10 with aSAH-WCI, and nine with UIA, were subjected to metagenomic sequencing and untargeted metabolomics profiling (Figure 1).

Fresh fecal samples (3–5 g) were collected into a sterile frozen tube (QIAGEN) in the morning using a clean sterile spoon, and the tube was then sealed tightly to create an anaerobic environment; the samples were transported to the laboratory using dry ice and stored in a −80 °C ultralow temperature freezer until further processing. For serum isolation, venous blood was collected using a serum separator tube and allowed to clot for 30 min before centrifugation for 10 min at approximately 3,000 × *g*. The supernatant serum was aliquoted and stored at −80 °C, avoiding repeated freeze-thaw cycles.

### 2.4 Metagenomic sequencing

Total microbial genomic DNA was extracted from fecal samples, and metagenomic shotgun sequencing libraries were constructed and sequenced on the DNBSEQ-T7 platform (Personal Biotechnology Co., Ltd., Shanghai, China). High-quality reads, filtered for host contamination, were subjected to taxonomic classification using Kraken2 against a customized GTDB database and assembled via MEGAHIT. Functional annotation of non-redundant genes was performed against the Kyoto Encyclopedia of Genes and Genomes (KEGG) database. Comprehensive bioinformatic pipelines and specific algorithm parameters are detailed in the Supplementary Method 2.1.

### 2.5 Untargeted metabolomics profiling

Fecal metabolites were extracted and subsequently profiled via liquid chromatography-mass spectrometry (LC-MS) analysis using an ACQUITY UPLC HSS T3 column coupled with a Thermo Orbitrap Exploris 120 mass spectrometer in both positive and negative HESI modes. Raw data alignment, peak extraction, and normalization were processed using MS-DIAL, with metabolites identified across comprehensive spectral libraries including mzCloud, HMDB, and LIPID MAPS. Full analytical parameters and quality control validations are described in the Supplementary Method 2.2.

### 2.6 Serum biomarker testing

The serum concentrations of inflammatory cytokines (IFN-γ, IL-1β, IL-6, IL-10, IL-18, TNF-α) and apoptosis indicators (BAX, Caspase-3, Cyt-C) were determined using specific enzyme-linked immunosorbent assay (ELISA) kits. Additionally, oxidative stress indicators, including malondialdehyde (MDA), superoxide dismutase (SOD), and glutathione (GSH), were quantified using corresponding microplate assay kits via the TBA, WST-8, and DTNB methods, respectively. All assays were performed strictly following the manufacturers’ protocols. Detailed operational procedures, including incubation times and absorbance measurement parameters, are described in the Supplementary Method 2.3.

### 2.7 Statistical analyses

All statistical procedures were implemented using R software (version 4.5.2; R Foundation for Statistical Computing, Vienna, Austria). For quantitative data following a normal distribution, comparisons between two groups were performed using an independent samples *t*-test, while comparisons among multiple groups were performed using one-way analysis of variance (ANOVA) combined with Tukey’s *post-hoc* test. For quantitative data not following a normal distribution, comparisons between two groups were performed using the Wilcoxon rank-sum test, while comparisons among multiple groups were performed using the Kruskal-Wallis H test combined with Dunn’s *post-hoc* test and false discovery rate (FDR) correction. For data corresponding to the categorical variables, a Pearson χ^2^ test was performed; if significant differences were found among multiple groups, subsequent pairwise comparisons were performed utilizing χ^2^ tests coupled with Bonferroni correction. Furthermore, to validate the adequacy of our sample size for the core multi-omics findings, a *post hoc* power analysis was conducted (Supplementary Method 3.1). To control for baseline confounding factors, inter-group comparisons were performed using Analysis of Covariance (ANCOVA), followed by *post-hoc* tests based on Estimated Marginal Means (EMMs) and FDR correction (Supplementary Method 3.2). To account for potential selection bias, multivariable sensitivity analysis was performed using ANCOVA, followed by *post-hoc* tests based on EMMs and FDR correction (Supplementary Method 3.3). Bioinformatics analyses, including linear discriminant analysis effect size (LEfSe), KEGG pathway enrichment, and partial least squares-discriminant analysis (PLS-DA), were employed to assess the differential signatures among the groups. Species-function contribution assessments and Spearman correlation analyses were utilized to evaluate the associations across these findings, facilitating the construction of an integrated correlation network encompassing gut microbiota, metabolites, and serum biomarkers. Finally, univariate receiver operating characteristic (ROC) curve analysis and leave-one-out cross-validation (LOOCV) were conducted, and the corresponding area under the curve (AUC) was calculated. Detailed bioinformatics methods were provided in the Supplementary Method 4.

## 3 Results

### 3.1 Clinical and demographic characteristics of the study cohort

The baseline demographic characteristics, comorbidities, and aneurysm characteristics were compared separately in the metagenomic, metabolomic, and serum biomarker cohorts. Although no significant differences were observed in baseline characteristics among the three groups in the metagenomic and metabolomic cohorts, significant differences were observed in the prevalence of hypertension (*P* = 0.008) and related complications (*P* = 0.023) in the serum biomarker cohort (Table 1). Further pairwise comparisons indicated that these differences were primarily attributable to the distinct distributions between the aSAH-CI and UIA groups (hypertension: *P* = 0.003; complications: *P* = 0.009).

**TABLE 1:** The basic clinical characteristics of aSAH-CI, aSAH-WCI, and UIA groups.

	Fecal omics cohort				Serum marker cohort			
Characteristics	aSAH-CI	aSAH-WCI	UIA	*P*-value	aSAH-CI	aSAH-WCI	UIA	*P*-value
*n*	11	10	9		18	15	15	
Sex, *n* (%)				0.342				0.520
Male	6 (54.5%)	5 (50.0%)	2 (22.2%)	–	9 (50.0%)	5 (33.3%)	5 (33.3%)	–
Female	5 (45.5%)	5 (50.0%)	7 (77.8%)	–	9 (50.0%)	10 (66.7%)	10 (66.7%)	–
Age, mean ± SD	59.27 ± 3.00	56.70 ± 4.74	54.44 ± 4.77	0.050	55.83 ± 6.16	53.93 ± 7.07	53.00 ± 5.26	0.412
BMI, *n* (%)				1				0.211
Underweight	1 (9.1%)	0 (0.0%)	0 (0.0%)	–	1 (5.6%)	0 (0.0%)	0 (0.0%)	–
Healthy weight	5 (45.5%)	4 (40.0%)	4 (44.4%)	–	9 (50.0%)	5 (33.3%)	6 (40.0%)	–
Overweight	4 (36.4%)	5 (50.0%)	5 (55.6%)	–	7 (38.9%)	6 (40.0%)	9 (60.0%)	–
Obesity	1 (9.1%)	1 (10.0%)	0 (0.0%)	–	1 (5.6%)	4 (26.7%)	0 (0.0%)	–
Education, *n* (%)				0.165				0.085
≤Primary	5 (45.5%)	4 (40.0%)	2 (22.2%)	–	8 (44.4%)	5 (33.3%)	3 (20.0%)	–
Junior high	3 (27.3%)	1 (10.0%)	4 (44.4%)	–	6 (33.3%)	5 (33.3%)	8 (53.3%)	–
Senior high	0 (0.0%)	2 (20.0%)	3 (33.3%)	–	0 (0.0%)	2 (13.3%)	4 (26.7%)	–
≥College	3 (27.3%)	3 (30.0%)	0 (0.0%)	–	4 (22.2%)	3 (20.0%)	0 (0.0%)	–
Smoking, *n* (%)				1				0.929
No	8 (72.7%)	7 (70.0%)	7 (77.8%)	–	12 (66.7%)	11 (73.3%)	10 (66.7%)	–
Yes	3 (27.3%)	3 (30.0%)	2 (22.2%)	–	6 (33.3%)	4 (26.7%)	5 (33.3%)	–
Drinking alcohol, *n* (%)				1				1
No	9 (81.8%)	8 (80.0%)	8 (88.9%)	–	14 (77.8%)	12 (80.0%)	12 (80.0%)	–
Yes	2 (18.2%)	2 (20.0%)	1 (11.1%)	–	4 (22.2%)	3 (20.0%)	3 (20.0%)	–
Hypertension, *n* (%)				0.242				0.008
No	2 (18.2%)	3 (30.0%)	5 (55.6%)	–	3 (16.7%)	4 (26.7%)	10 (66.7%)	–
Yes	9 (81.8%)	7 (70.0%)	4 (44.4%)	–	15 (83.3%)	11 (73.3%)	5 (33.3%)	–
Diabetes, *n* (%)				0.899				0.666
No	6 (54.5%)	6 (60.0%)	6 (66.7%)	–	12 (66.7%)	11 (73.3%)	12 (80.0%)	–
Yes	5 (45.5%)	4 (40.0%)	3 (33.3%)	–	6 (33.3%)	4 (26.7%)	3 (20.0%)	–
Dyslipidemia, *n* (%)				0.633				0.625
No	11 (100.0%)	9 (90.0%)	9 (100.0%)	–	18 (100.0%)	14 (93.3%)	15 (100.0%)	–
Yes	0 (0.0%)	1 (10.0%)	0 (0.0%)	–	0 (0.0%)	1 (6.7%)	0 (0.0%)	–
Hunt—Hess, *n* (%)				0.857				–
I	3 (27.3%)	3 (30.0%)	–	–	3 (16.7%)	4 (26.7%)	–	–
II	6 (54.5%)	4 (40.0%)	–	–	8 (44.4%)	5 (33.3%)	–	–
III	2 (18.2%)	3 (30.0%)	–	–	7 (38.9%)	6 (40.0%)	–	–
Fisher, *n* (%)				1				0.571
1	2 (18.2%)	2 (20.0%)	–	–	2 (11.1%)	3 (20.0%)	–	–
2	7 (63.6%)	6 (60.0%)	–	–	11 (61.1%)	8 (53.3%)	–	–
3	1 (9.1%)	0 (0.0%)	–	–	2 (11.1%)	0 (0.0%)	–	–
4	1 (9.1%)	2 (20.0%)	–	–	3 (16.7%)	4 (26.7%)	–	–
Complications, *n* (%)				0.309				0.023
No	7 (63.6%)	9 (90.0%)	8 (88.9%)	–	9 (50.0%)	11 (73.3%)	14 (93.3%)	–
Yes	4 (36.4%)	1 (10.0%)	1 (11.1%)	–	9 (50.0%)	4 (26.7%)	1 (6.7%)	–
Aneurysm								
Size, mean ± SD	8.65 ± 3.47	7.04 ± 2.30	6.63 ± 1.93	0.221	9.24 ± 3.40	7.61 ± 2.36	7.04 ± 2.13	0.064
Height-width ratio, mean ± SD	1.85 ± 0.57	1.70 ± 0.58	1.42 ± 0.34	0.207	1.78 ± 0.47	1.67 ± 0.51	1.53 ± 0.30	0.253
Location, *n* (%)				1				1
Anterior circulation	8 (72.7%)	8 (80.0%)	7 (77.8%)	–	15 (83.3%)	13 (86.7%)	13 (86.7%)	–
Posterior circulation	3 (27.3%)	2 (20.0%)	2 (22.2%)	–	3 (16.7%)	2 (13.3%)	2 (13.3%)	–
Arterial bifurcation, *n* (%)				1				1
No	9 (81.8%)	8 (80.0%)	8 (88.9%)	–	16 (88.9%)	13 (86.7%)	13 (86.7%)	–
Yes	2 (18.2%)	2 (20.0%)	1 (11.1%)	–	2 (11.1%)	2 (13.3%)	2 (13.3%)	–
Multiple aneurysms, *n* (%)				0.224				1
No	8 (72.7%)	7 (70.0%)	9 (100.0%)	–	14 (77.8%)	12 (80.0%)	11 (73.3%)	–
Yes	3 (27.3%)	3 (30.0%)	0 (0.0%)	–	4 (22.2%)	3 (20.0%)	4 (26.7%)	–
PPIs, *n* (%)				0.428				0.288
No	4 (36.4%)	4 (40.0%)	6 (66.7%)	–	8 (44.4%)	6 (40.0%)	10 (66.7%)	–
Yes	7 (63.6%)	6 (60.0%)	3 (33.3%)	–	10 (55.6%)	9 (60.0%)	5 (33.3%)	–
Opioids, *n* (%)				0.637				0.559
No	6 (54.5%)	6 (60.0%)	7 (77.8%)	–	10 (55.6%)	9 (60.0%)	11 (73.3%)	–
Yes	5 (45.5%)	4 (40.0%)	2 (22.2%)	–	8 (44.4%)	6 (40.0%)	4 (26.7%)	–
Laxatives, *n* (%)				1				0.932
No	6 (54.5%)	5 (50.0%)	5 (55.6%)	–	10 (55.6%)	8 (53.3%)	9 (60.0%)	–
Yes	5 (45.5%)	5 (50.0%)	4 (44.4%)	–	8 (44.4%)	7 (46.7%)	6 (40.0%)	–

Baseline comparisons between the cohort with available fecal samples (*n* = 30) and the excluded cohort lacking fecal data (*n* = 18) revealed selection bias regarding age, diabetes prevalence, and the proportion of posterior circulation aneurysms (Supplementary Table 1).

### 3.2 Metagenomic data demonstrated taxonomic signatures of gut microbiota in aSAH-CI, aSAH-WCI, and UIA groups

**FIGURE 2:**
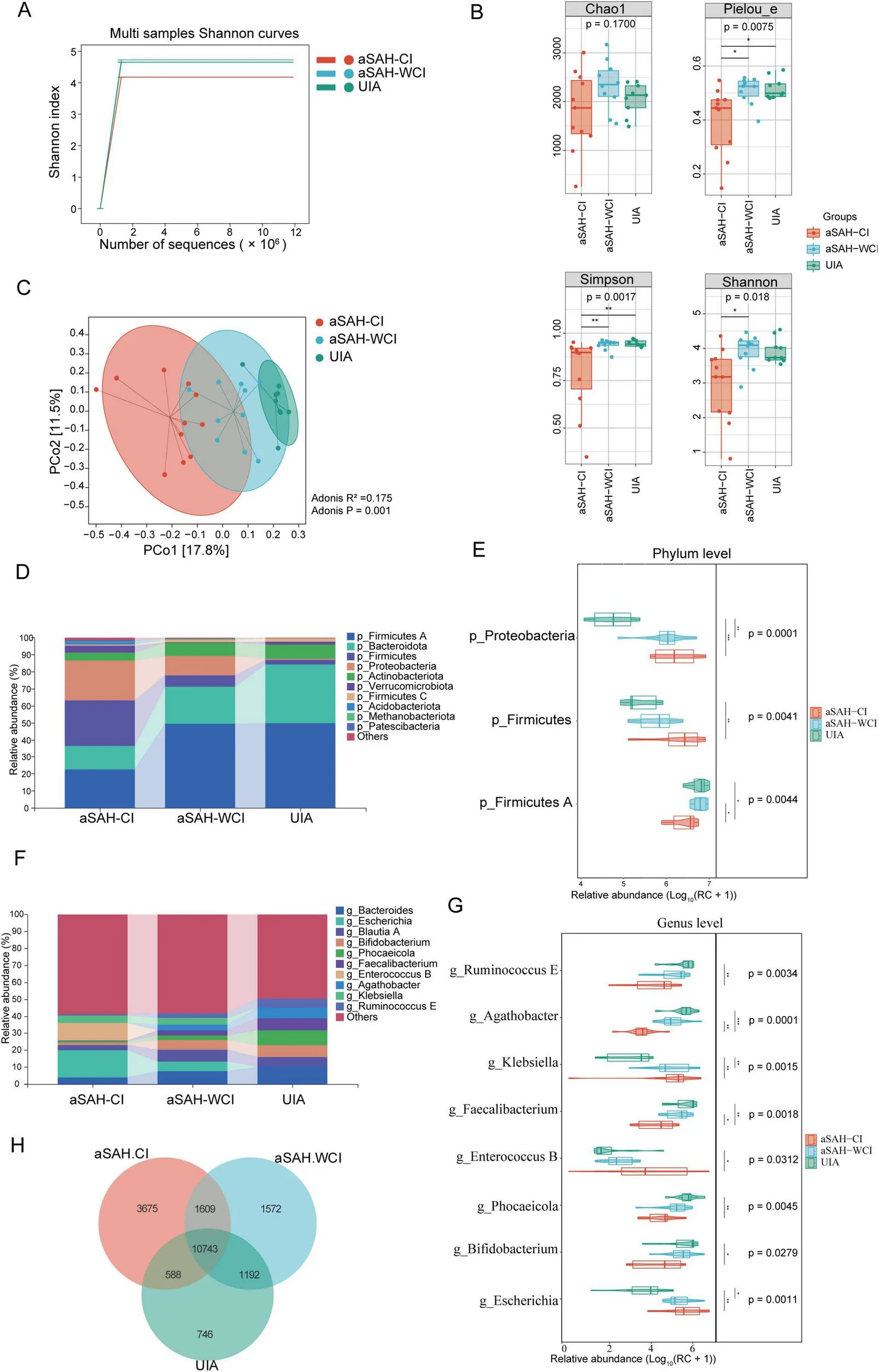
Taxonomic signatures and diversity alterations of the gut microbiota among the aSAH-CI, aSAH-WCI, and UIA groups. **(A)** Shannon diversity index curve utilized to evaluate the sufficiency of sample size and sequencing depth. **(B)** Box plots showing alpha diversity comparisons among all groups with Chao1 index, Pielou’s evenness index, Shannon index and Simpson index. **(C)** Principal Coordinate Analysis (PCoA) based on Bray-Curtis distances for beta diversity, indicating a different distribution among all groups. **(D)** Bar plots showing relative abundance of gut microbiota at the phylum level. **(E)** Box plots showing significant differences in the relative abundance of dominant phyla. **(F)** Bar plots showing the relative abundance of gut microbiota at the genus level. **(G)** Box plots showing significant differences in the relative abundance of dominant genera. **(H)** Venn diagram depicting the number of both shared and distinct species among the various groups. (*P*-value, *P < 0.05; **P < 0.01; ***P < 0.001).

The Shannon rarefaction curves plateaued, indicating that the sequencing depth was sufficient to capture the majority of the microbial communities (Figure 2A). Alpha diversity analysis revealed a significant decrease in gut microbial diversity in the aSAH-CI group compared with the aSAH-WCI group, evidenced by significant declines in both the Shannon and Simpson indices (*P* < 0.05). Although no significant difference was observed in species richness measured by the Chao1 index among the groups, the evenness of the microbial community measured by Pielou’s evenness index was significantly lower in the aSAH-CI group than in the other two groups (Figure 2B). Furthermore, beta diversity analysis based on Bray-Curtis distance revealed a significant separation in the gut microbial community structure among the three groups, which is supported by an Adonis analysis (Figure 2C, *R*^2^ = 0.175, *P* = 0.001). Notably, the community structure of the aSAH-CI group diverged significantly from those of the aSAH-WCI and UIA groups (*P* < 0.05), suggesting that patients who later develop cognitive impairment following aSAH possess a unique acute-phase microecological profile.

Regarding taxonomic composition, the groups were dominated at the phylum level by Firmicutes A, Bacteroidota, Firmicutes, Proteobacteria, and Actinobacteriota (Figure 2D). Compared to the aSAH-WCI and UIA groups, the abundance of Firmicutes A in the aSAH-CI group was significantly decreased (*P* < 0.05), whereas the abundances of Firmicutes and Proteobacteria were increased (Figure 2E). At the genus level, the relative abundances of the putative commensal bacteria *Faecalibacterium* and *Agathobacter* were significantly lower in the aSAH-CI group compared to the aSAH-WCI and UIA groups (*P* < 0.05), with no statistical difference observed between the latter two groups. Furthermore, *Bifidobacterium*, *Phocaeicola*, and *Ruminococcus E* also exhibited progressive depletion in the aSAH-CI group, whereas the abundances of potential opportunistic pathogens, including *Escherichia*, *Klebsiella*, and *Enterococcus B*, increased (Figures 2F, G).

**FIGURE 3:**
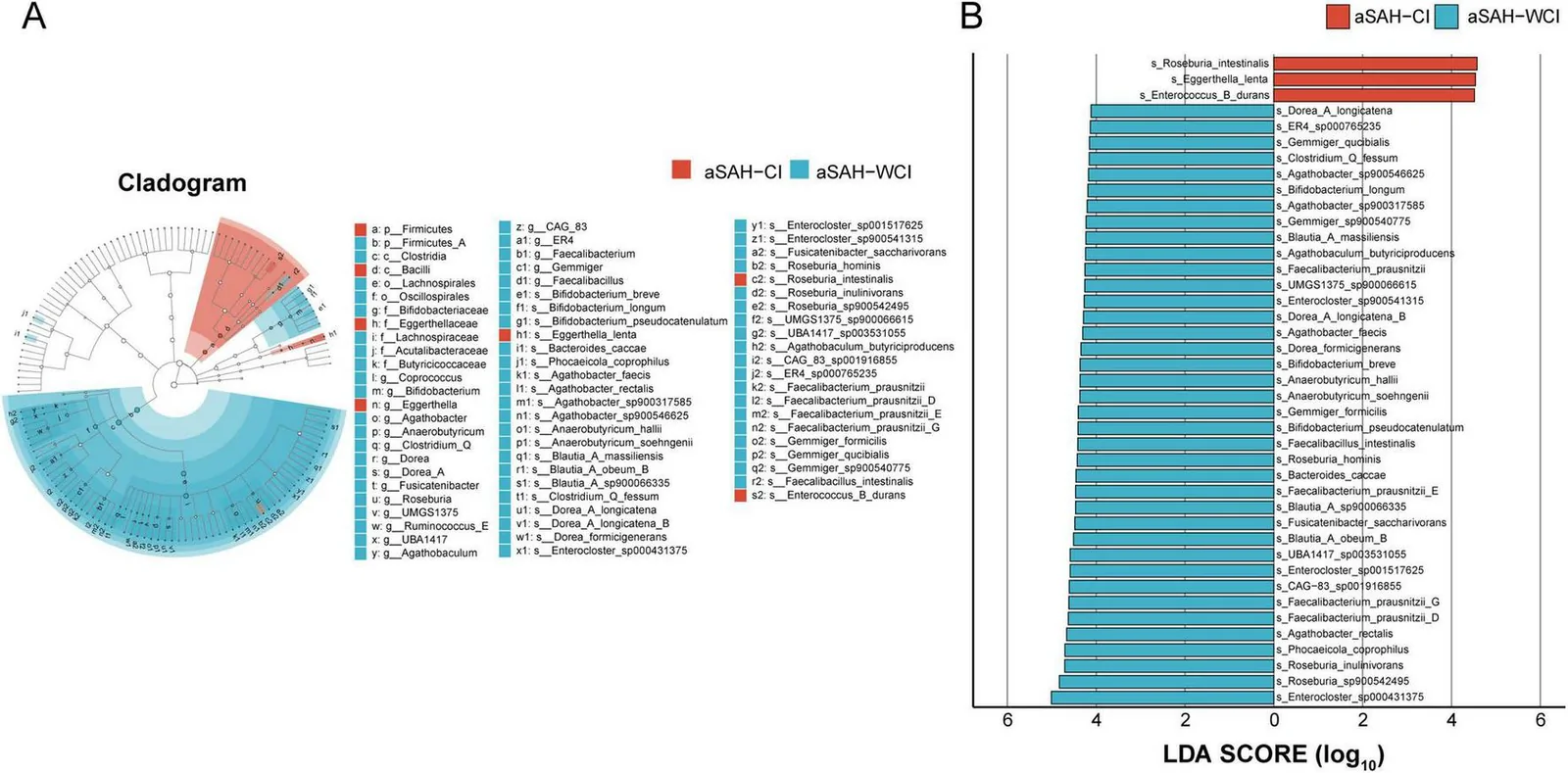
The significant differences in gut microbiota characteristics between aSAH-CI and aSAH-WCI groups. **(A)** LEfSe cladogram visualizing the phylogenetic distribution of differentially abundant taxa between the aSAH-CI and aSAH-WCI groups. **(B)** LDA bar plots showing 41 key differential species (LDA score > 2.0).

To accurately identify the characteristic species across the groups, Venn diagram analysis first identified 3,675, 1,572, and 746 specific species in the aSAH-CI, aSAH-WCI, and UIA groups, respectively (Figure 2H). Subsequently, LEfSe analysis (LDA > 2.0, *P* < 0.05) was employed for an in-depth exploration of the gut microbiota profiles between the aSAH-CI and aSAH-WCI groups. A taxonomic cladogram demonstrated the phylogenetic distribution of significantly different microbial communities across various taxonomic levels (Figure 3A), while the LDA bar chart further quantified the effect sizes of 41 key differentially abundant bacterial species at the species level (Figure 3B). In the acute-phase samples of the aSAH-CI group, only a few species, such as *Eggerthella lenta* and *Enterococcus B durans*, were abnormally enriched; conversely, the abundances of numerous previously reported beneficial species, including *Agathobacter rectalis*, *Roseburia inulinivorans*, and *Phocaeicola coprophilus*, were significantly decreased. This profound remodeling of taxonomic signatures represents a critical microbiological feature closely associated with the clinical manifestation of cognitive impairment following aSAH.

### 3.3 Functional metagenomic profiling reveals the acute-phase depletion of amino acid metabolism pathways associated with subsequent aSAH-CI

**FIGURE 4:**
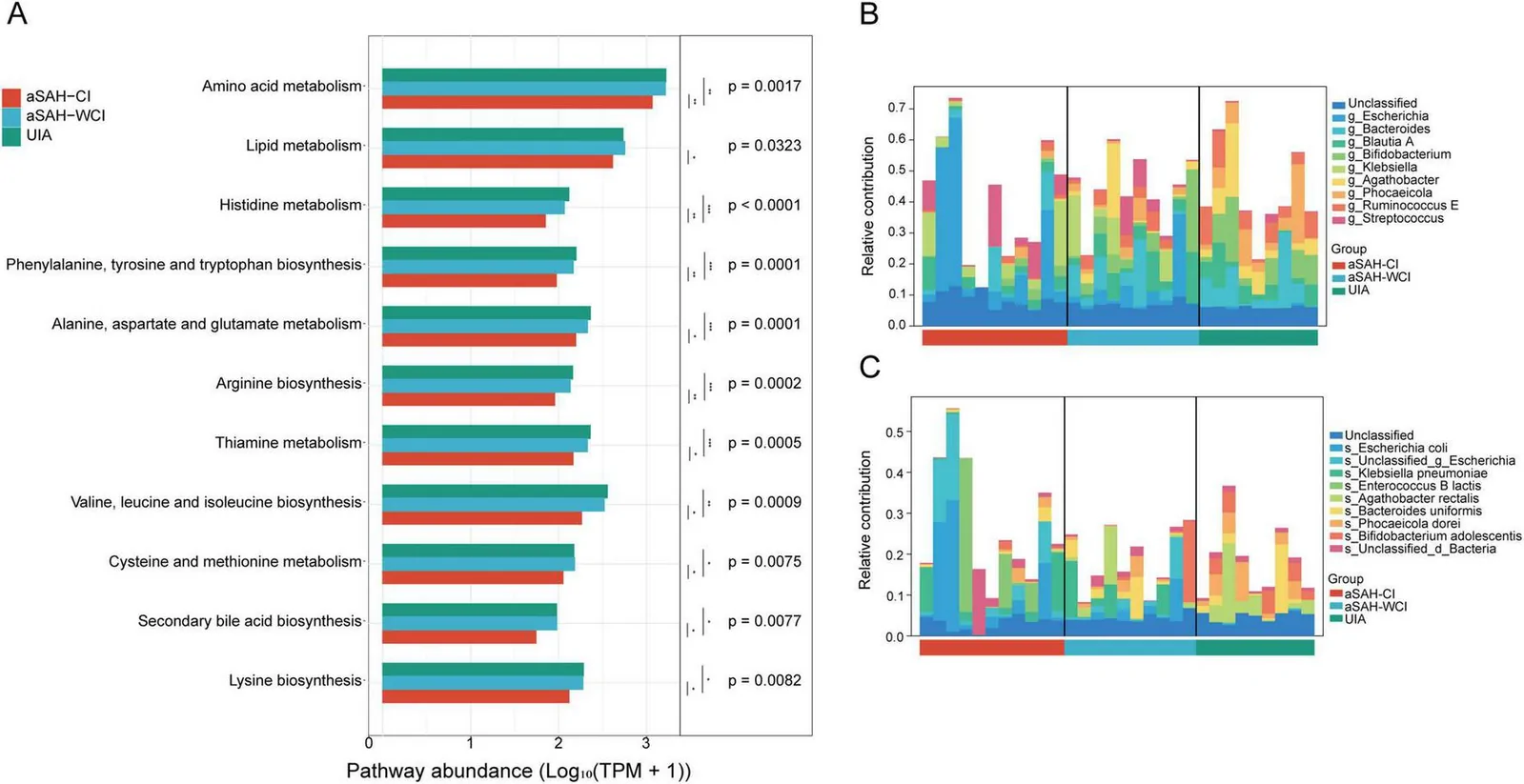
Functional metagenomic profiling reveals alterations in amino acid biosynthesis pathways. **(A)** Bar plots showing significant differential metabolic pathways among all groups. **(B)** Genus-level contribution analysis of the amino acid metabolism pathway. **(C)** Species-level contribution analysis of the phenylalanine, tyrosine and tryptophan biosynthesis pathway. (*P*-value, *P < 0.05; **P < 0.01; ***P < 0.001).

Functional profiling of the metagenomes using the KEGG database identified significant alterations in microbial metabolic capacities among the three groups (Supplementary Figures 1A–C). Specifically, at the KEGG L2 level, the abundance of pathways associated with amino acid metabolism was significantly reduced in the acute-phase microbiomes of the aSAH-CI group relative to the aSAH-WCI and UIA cohorts (*P* < 0.05). Similarly, lipid metabolism was also significantly less abundant compared to the aSAH-WCI group (*P* < 0.05). Deeper functional characterization at the L3 level demonstrated that multiple key amino acid metabolic sub-pathways were significantly depleted in the aSAH-CI microbiome, including histidine metabolism; phenylalanine, tyrosine, and tryptophan biosynthesis; alanine, aspartate, and glutamate metabolism; and arginine biosynthesis (Figure 4A, *P* < 0.05). Notably, these pathways exhibited no significant differences between the aSAH-WCI and UIA groups.

To further elucidate the biological origins of the altered gut functional profiles in patients with aSAH-CI, this study employed species-function contribution analysis to map the differential KEGG metabolic pathways to specific microbial taxa. The results indicated that within the significantly downregulated amino acid metabolism pathway at the L2 level, the genus *Agathobacter* was the core functional contributor to this pathway (Figure 4B). Further in-depth analysis at the species level confirmed that *Agathobacter rectalis*, a significantly depleted bacterial species identified in the previous LEfSe analysis, was identified as a major contributor to the predicted abundance of key pathways, such as phenylalanine, tyrosine, and tryptophan biosynthesis (Figure 4C). These findings strongly suggest that the depletion of *Agathobacter rectalis* in the acute-phase gut of patients who subsequently developed aSAH-CI is highly correlated with alterations in the synthesis pathways for critical neurometabolic precursors, including aromatic amino acids. This dynamic may represent a crucial metabolic correlate linked to the progression of the disease, warranting further investigation.

### 3.4 Fecal metabolomics reveals a profound acute-phase shift from amino acid synthesis to neurotoxic trace amine accumulation associated with later aSAH-CI

**FIGURE 5:**
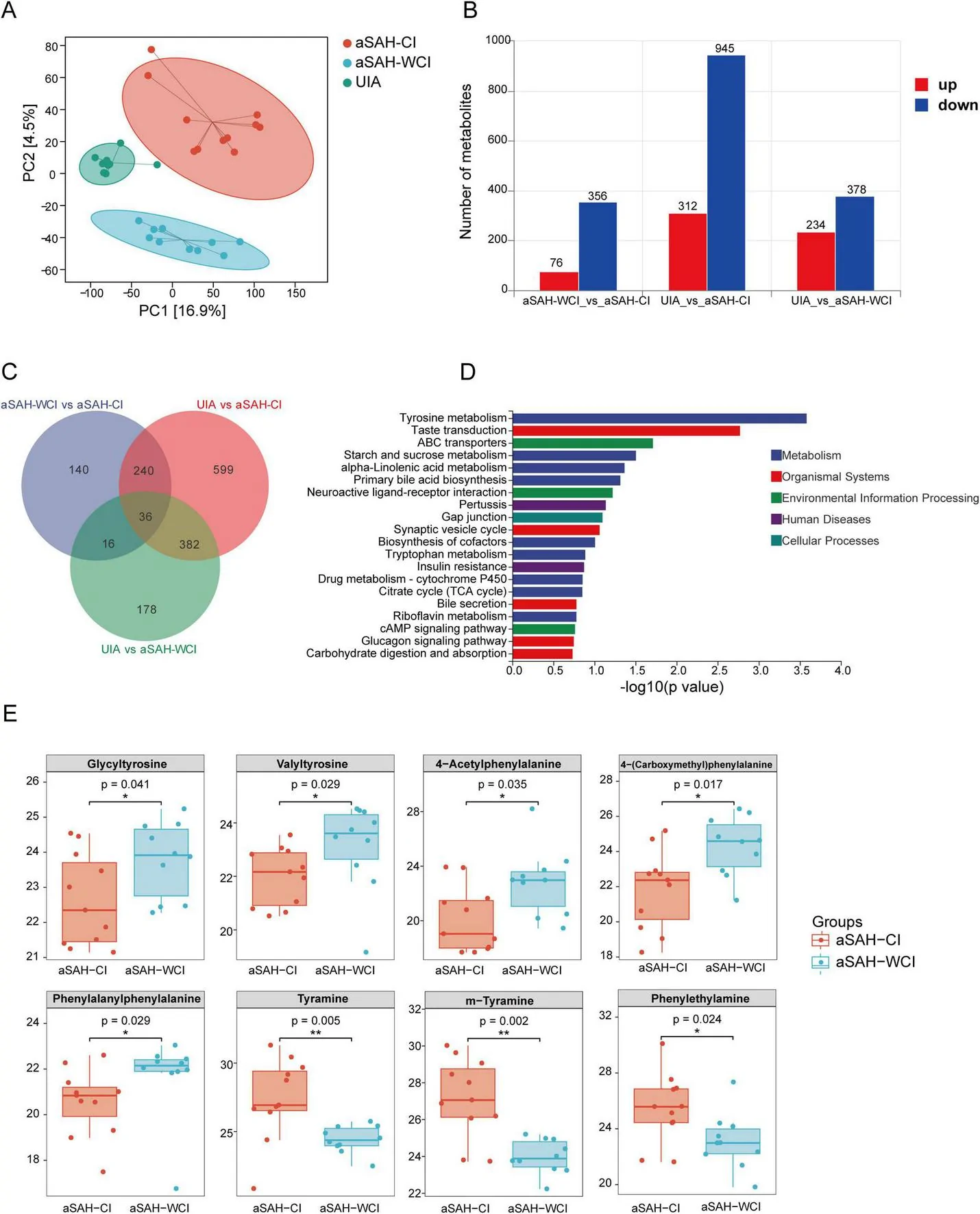
Identification of differential metabolites among aSAH-CI, aSAH-WCI, and UIA groups. **(A)** PLS-DA score plot illustrating a distinct spatial separation of overall metabolic profiles among the three groups. **(B,C)** Bar plots and Venn diagram showing the number of differential metabolites identified. **(D)** KEGG enrichment analysis for differential metabolites between aSAH-CI and aSAH-WCI groups. **(E)** Box plots showing comparisons of eight selected differential metabolites between aSAH-CI and aSAH-WCI groups. (*P*-value, *P < 0.05; **P < 0.01).

The remodeling of gut microecology and functional pathways is frequently accompanied by significant alterations to downstream metabolite profiles. Consequently, we conducted an untargeted metabolomics analysis on the fecal samples. An overall substance clustering heatmap preliminarily delineated the differences in metabolite signatures among patients in the aSAH-CI, aSAH-WCI, and UIA groups (Supplementary Figures 1D, E). PLS-DA demonstrated a significant spatial separation in the overall metabolic profiles among the three groups, confirming the existence of distinct gut metabolic microenvironments among patients with different cognitive outcomes following aSAH (Figure 5A). Furthermore, the PLS-DA permutation test indicated that the model possessed robust explanatory and predictive capabilities for the categorical variables (Supplementary Figure 1F, *R*^2^*Y* = 0.996, *Q*^2^ = 0.776).

After elucidating the overall differences in the metabolic profiles, we further explored the differential metabolites among the groups. Using FDR-corrected *P*-value < 0.05 and a variable importance in projection (VIP) score > 1 as the screening criteria, the results revealed 432 and 1,257 differential metabolites in the aSAH-CI group compared to the aSAH-WCI and UIA groups, respectively, including 276 shared core differential metabolites (Figures 5B, C). To investigate the association between gut metabolites and cognitive impairment, we conducted a KEGG pathway enrichment analysis on the differential metabolites between the aSAH-CI and aSAH-WCI groups. The study revealed that tyrosine metabolism, starch and sucrose metabolism, alpha-linolenic acid metabolism, and primary bile acid biosynthesis were the predominantly enriched metabolic pathways (Figure 5D). These findings at the metabolomic level are consistent with the findings on amino acid metabolism and lipid metabolism discussed earlier in the metagenomics section. Furthermore, they provide a high degree of cross-omics corroboration for the phenylalanine, tyrosine, and tryptophan biosynthesis pathway.

Based on the results of the KEGG enrichment analysis, differential metabolites associated with tyrosine metabolism between the aSAH-CI and aSAH-WCI groups were selected for presentation. Compared to the aSAH-WCI group, the levels of core neurotransmitter precursors (including phenylalanine, tyrosine, and their derivatives) in the acute-phase feces of patients who developed aSAH-CI were significantly decreased; conversely, trace amines, which are amino acid decarboxylation products with potential neurovascular toxicity (such as tyramine and phenylethylamine), were significantly elevated (Figure 5E).

### 3.5 Associations between the acute-phase gut microbiota and metabolites related to later aSAH-CI

**FIGURE 6:**
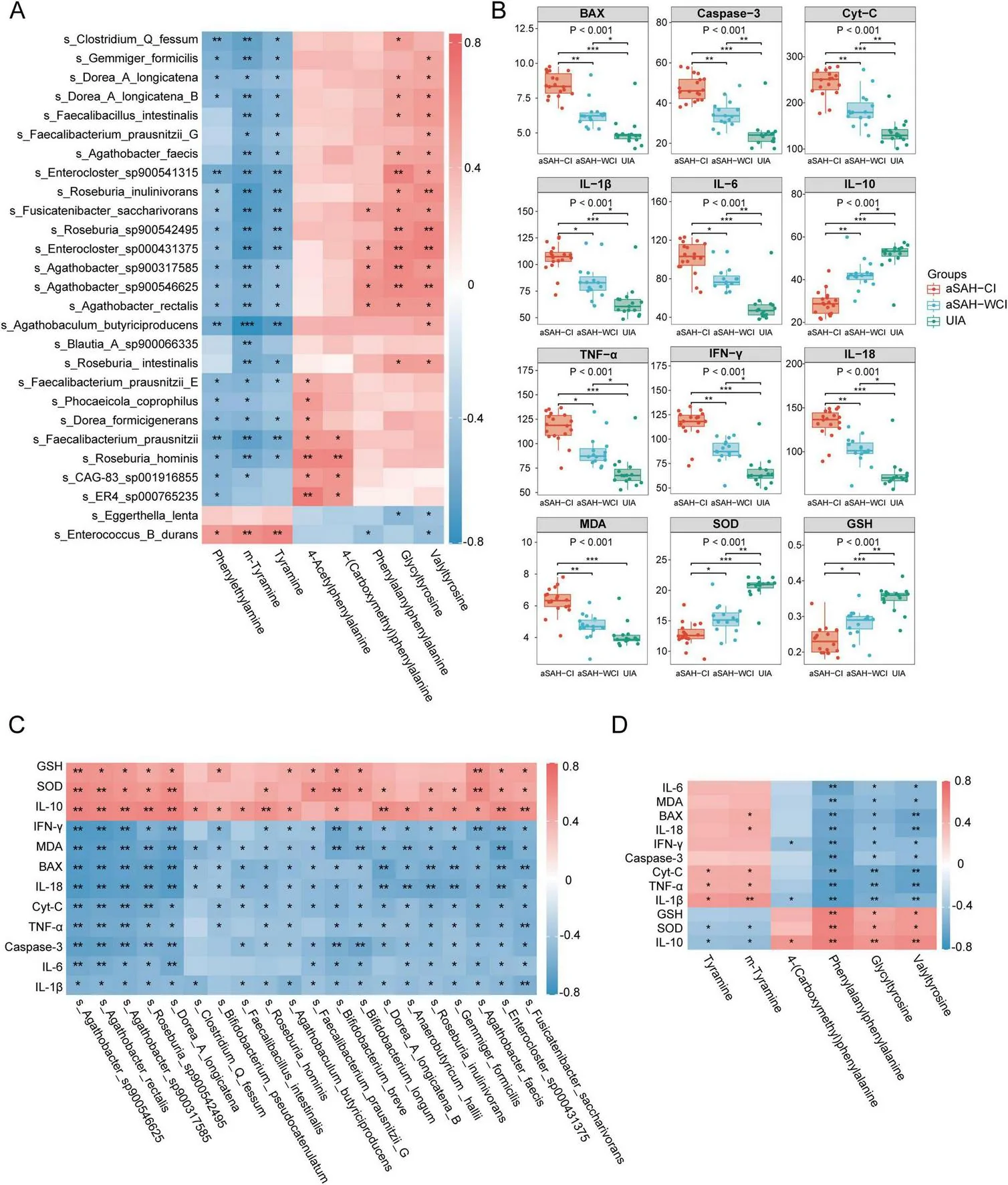
The correlations among gut microbiota, fecal metabolites and host serum biomarkers. **(A)** Heatmap detailing the correlations between gut microbiota and metabolites. **(B)** Box plots showing comparisons of serum biomarkers among the aSAH-CI, aSAH-WCI, and UIA groups. **(C)** Heatmap detailing the correlations between gut microbiota and serum biomarkers. **(D)** Heatmap detailing the correlations between fecal metabolites and serum biomarkers. (*P*-value, *P < 0.05; **P < 0.01; ***P < 0.001).

To further investigate the interactive network between gut microecological remodeling and metabolite variations in cognitive impairment following aSAH, we performed a Spearman correlation analysis between key differential microbiota and core metabolites (Figure 6A). The correlation matrix revealed two distinctly different “microbiota-metabolite” covariation patterns. The results indicated that trace amines with potential neurovascular toxicity (such as tyramine, m-tyramine, and phenylethylamine) exhibited a significant positive correlation with the opportunistic pathogen (*Enterococcus B durans*) that was significantly enriched in the aSAH-CI group (*P* < 0.05). Conversely, these putative neurotoxic amine metabolites exhibited a significant negative correlation with the beneficial commensal microbiota enriched in the aSAH-WCI group (such as *A. rectalis*) (*P* < 0.05). Furthermore, amino acids and their derivatives, which serve as crucial precursors for neurotransmitter synthesis (such as glycyltyrosine and valyltyrosine), displayed a completely opposite pattern in their abundance variations: they were negatively correlated with the pathogenic strains enriched in the aSAH-CI group and positively correlated with the beneficial microbiota associated with the aSAH-WCI group.

### 3.6 Comparison of serum biomarkers in aSAH-CI, aSAH-WCI, and UIA groups

To evaluate the association between gut microecological remodeling and the overall pathophysiological state of the host, we compared serum markers of inflammation, oxidative stress, and apoptosis among the three groups of patients. After statistically adjusting for baseline clinical confounders (hypertension and complications) via ANCOVA, the results demonstrated that patients who eventually developed aSAH-CI were in an acute state of significant systemic inflammatory activation, oxidative stress, and apoptotic imbalance (Figure 6B). Specifically, the expression levels of pro-inflammatory cytokines (IFN-γ, IL-1β, IL-6, IL-18, and TNF-α), a lipid peroxidation marker (MDA), and pro-apoptotic indices (BAX, Caspase-3, and Cyt-C) in the aSAH-CI group were significantly higher than those in the aSAH-WCI and UIA groups (*P* < 0.05). Conversely, the reserves of an anti-inflammatory cytokine (IL-10) and core antioxidants (SOD and GSH) in this group of patients were significantly depleted (*P* < 0.05).

To investigate the potential link between the aforementioned host systemic pathophysiological state and alterations in the gut microenvironment, we performed a comprehensive correlation analysis of “serum markers-gut microecology (microbiota/metabolites).” At the microbiomic level, these serum phenotypes exhibited no correlation with the pathogenic microbiota significantly enriched in the aSAH-CI group (*E. lenta*, *Enterococcus* B *durans*). Conversely, the beneficial commensal bacteria enriched in the aSAH-WCI group (such as *A. rectalis*) demonstrated a distinct pattern associated with systemic homeostasis; specifically, they were positively correlated with anti-inflammatory and antioxidant indices, and negatively correlated with pro-inflammatory and pro-apoptotic markers (Figure 6C). Notably, the accumulation of trace amines with potential neurovascular toxicity (such as tyramine) not only co-occurred with the abundance of pathogenic microbiota but was also significantly positively correlated with the elevation of pro-inflammatory and pro-apoptotic factors, as well as the attenuation of the antioxidant defense system in the host serum. Conversely, the maintenance of levels of key amino acids and their derivatives that serve as neurotransmitter precursors (such as tyrosine and phenylalanine) was significantly positively correlated with a lower inflammatory burden and superior antioxidant capacity in the body (Figure 6D).

### 3.7 Combined multi-omics analysis of differential gut microbiota, metabolites, and serum biomarkers

**FIGURE 7:**
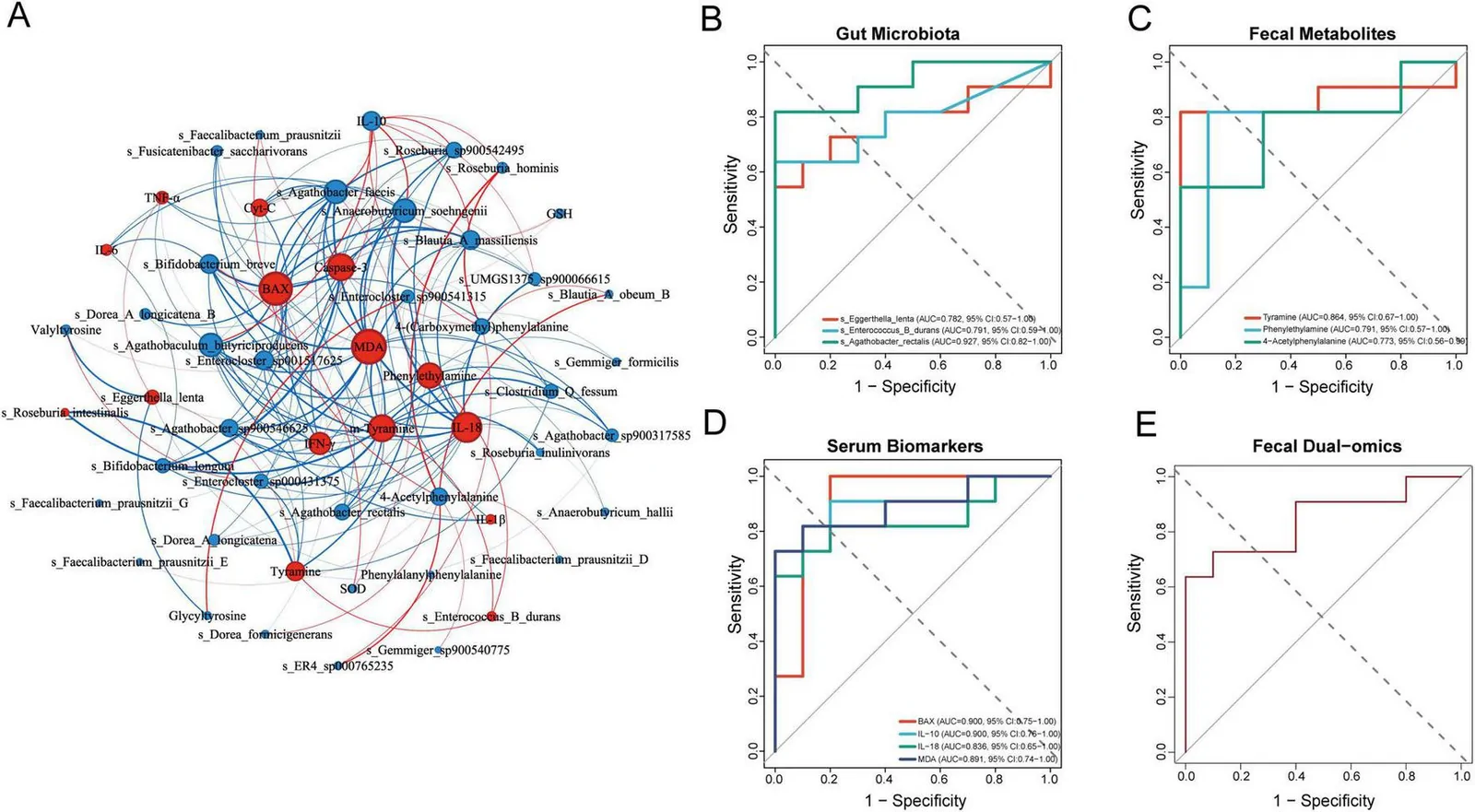
Integrated cross-omics network and the evaluation of multi-dimensional biomarkers for early diagnosis. **(A)** Cross-omics Spearman correlation network integrating differential gut microbiota, fecal metabolites, and serum biomarkers. Node size is proportional to degree centrality. Red and blue circles indicate enrichment in the aSAH-CI and aSAH-WCI groups, respectively. Red and blue lines represent positive and negative correlations, respectively. **(B–D)** ROC curves evaluating the clinical diagnostic value of the multi-dimensional biomarkers. **(E)** LOOCV-adjusted ROC curve of the fecal dual-omics panel.

To systematically evaluate the interactive network among cross-omics features, we constructed a Spearman correlation network encompassing differential microbiota, metabolites, and serum markers (Figure 7A). Topological analysis based on degree centrality revealed two densely negatively correlated functional modules: in the aSAH-CI module, systemic injury markers (BAX, MDA, IL-18) and a putative neurovascular toxic metabolite (phenylethylamine) constituted the hub nodes; whereas the aSAH-WCI module featured an anti-inflammatory cytokine (IL-10) and an amino acid derivative (4-acetylphenylalanine) as key homeostatic nodes.

Based on the aforementioned hub features and significantly differential markers among the groups, univariate ROC analysis confirmed their potential value in the early diagnosis of cognitive impairment following aSAH. Markers across multiple dimensions demonstrated extremely high predictive efficacy: at the microecological level, the depletion of *A. rectalis* (AUC = 0.927, 95% CI: 0.82–1.00) and the enrichment of species such as *E. lenta* held significant diagnostic value (Figure 7B); at the metabolic level, the abnormal elevation of tyramine also exhibited robust predictive power (Figure 7C, AUC = 0.864, 95% CI: 0.67–1.00); at the host serum level, the elevations of BAX and MDA, as well as the reduction of IL-10, all demonstrated excellent sensitivity and specificity (Figure 7D, all AUCs ≥ 0.891). To mitigate model overfitting and adhere to the statistical dimensionality constraints of this restricted cohort, LOOCV was performed to evaluate a non-invasive, gut-microbiome-derived multi-omics panel consisting solely of the core depleted bacterium *A. rectalis* and the enriched putative neurotoxic metabolite tyramine. Following internal cross-validation, this combined predictive model achieved an AUC of 0.845 (Figure 7E, 95% CI: 0.669–1.000). These data indicate that integrating multi-dimensional marker signatures possesses significant translational potential for early clinical warning.

## 4 Discussion

As research into the “gut-brain axis” continues to deepen, the association between gut microbiota dysbiosis and neurocognitive impairment has garnered widespread attention (Frileux et al., 2024; Liu et al., 2025; Warren et al., 2026). It is particularly noteworthy that the rapid emergence of various physiological changes in patients following aSAH collectively is closely linked to a state of gut microbiota dysbiosis. During the acute phase, the body’s stress response activates the sympathetic nervous system and the hypothalamic-pituitary-adrenal axis, which has been shown to affect gastrointestinal motility and secretory functions, ultimately altering the composition of the gut microbiota (Singh et al., 2016; Morais et al., 2020). Furthermore, the impairment of gut barrier function, coupled with iatrogenic factors such as the surgical procedure itself and the administration of antibiotics, has also been associated with alterations in the microbiota composition (Singh et al., 2016). However, the associations between gut microbiota alterations and cognitive impairment following aSAH remain poorly understood. Systematic research characterizing the comprehensive multi-omics correlative signatures and early predictive patterns of cognitive impairment in this patient population remains limited.

This study employed a multi-omics integrative analysis to explore the gut microbiota and metabolic profiles of patients, providing a systematic elucidation in conjunction with peripheral blood levels of oxidative stress and inflammation. Fan and Pedersen (2020) discovered that high diversity and high genetic richness are hallmarks of a healthy gut microbiota. The results of the present study demonstrated a loss of alpha diversity in the acute-phase gut microbiota of patients who progressed to aSAH-CI, indicating a decrease in species richness and evenness, which aligns with the gut microbiota characteristics previously observed in patients with cognitive impairment (Xi et al., 2021). Furthermore, in the beta diversity analysis, the Bray-Curtis distance revealed that the aSAH-CI group exhibited significant differences compared to the other groups. Against the backdrop of an overall abnormal microecological structure, this study further identified the characteristic changes of the core microbiota.

The study revealed a pathological enrichment of specific potential opportunistic pathogens, particularly *E. lenta* and *Enterococcus* B *durans*, in the acute-phase samples of the aSAH-CI group. While our observational data cannot establish a direct mechanism, previous studies have demonstrated that the abnormal expansion of these bacteria can increase the release of intestinal endotoxin lipopolysaccharide (LPS), which has been reported to disrupt the intestinal mucus layer barrier. Crucially, literature suggests it can also specifically activate the T helper 17 (Th17) cell immune response, thereby inducing systemic inflammation (Alexander et al., 2022; Zhang et al., 2023). The enrichment of such pathogenic bacteria represents a crucial microbiological signature accompanying the host’s progression from localized microecological dysbiosis to severe systemic inflammation following aSAH. Furthermore, the study revealed a significant reduction in putative butyrate-producing microbiota in the acute stage of patients with aSAH-CI compared to the aSAH-WCI and UIA groups, particularly a sharp decline in the abundances of species within the genera *Faecalibacterium* and *Agathobacter*. Notably, no significant difference was observed in these two genera between the aSAH-WCI and UIA groups, suggesting that their depletion may be specifically correlated with the development of cognitive impairment following aSAH. These microbiota are considered the cornerstones of gut health, and their metabolite, butyrate, has been widely documented to act as a crucial signaling molecule linking the gut and the brain to maintain the integrity of the intestinal mucosal barrier and the blood-brain barrier (BBB), thereby exerting established neuroprotective effects in the onset and progression of cognitive impairment (Koh et al., 2016; Pham et al., 2021).

Previous studies have often regarded the aforementioned depleted microbiota as butyrate-producing bacteria. The species-function contribution analysis in the present study suggested a potential underlying mechanism: these commensal bacteria are the primary predicted contributors to the amino acid biosynthesis pathways within the host gut (particularly phenylalanine, tyrosine, and tryptophan biosynthesis). Research has found that bacteria of the genus *Agathobacter* are involved in the metabolism of carbohydrates and amino acids (Wu et al., 2022). Our metagenomic functional annotation revealed a predicted loss of amino acid metabolism function in the acute phase of patients who subsequently developed aSAH-CI. Furthermore, no significant difference in amino acid metabolism was observed between the aSAH-WCI and UIA groups, suggesting that this depletion may represent a specific metabolic signature associated with the development of cognitive impairment following aSAH. In addition to amino acid metabolism, the present study also observed a significant decline in lipid metabolism in the aSAH-CI group compared to the aSAH-WCI group. The brain is a highly lipid-rich organ in the human body. Lipids are not only crucial components of neuronal cell membranes and myelin sheaths, but their associated metabolites (such as alpha-linolenic acid) also play a key neuroprotective role against neuroinflammation (Kim and Song, 2024; Sinclair, 2024). The acute-phase attenuation of lipid metabolism function in the gut microbiota of patients with aSAH-CI may potentially restrict the subsequent self-repair capacity of neurons following hemorrhagic toxic insults and secondary ischemia, and might affect the regeneration of myelin sheaths, thereby serving as a potential underlying metabolic correlate for the persistent decline in cognitive function at a deeper structural level (Kim and Song, 2024).

Furthermore, although our study cohort did not exhibit statistically significant sex differences due to the limited sample size, epidemiological evidence has established a higher prevalence of aSAH among females, particularly in postmenopausal populations (Molenberg et al., 2022). Sex hormones, predominantly estrogen, play a critical role in maintaining the integrity of the intestinal mucosal barrier and shaping a healthy microbiome (Pace and Watnick, 2021; Brettle et al., 2022). Estrogen not only promotes the proliferation of beneficial commensal bacteria but also exerts a protective effect against systemic neuroinflammation (Pace and Watnick, 2021; Brettle et al., 2022). Consequently, under the acute physiological stress induced by aSAH, patients with diminished estrogen-mediated protection may have a higher inherent susceptibility to the severe disruption of gut micro-ecology. This sex-driven susceptibility might be associated with the specific microbial dysbiosis observed in patients with aSAH-CI. Future studies with expanded sample sizes are warranted to conduct sex-stratified analyses, which will help control for sex-related confounding effects and further validate our findings regarding the microbiota-gut-brain axis.

The depletion of specific protective microbiota, the enrichment of pathogenic bacteria, and the associated alterations in metabolic pathways closely align with the positive findings of our untargeted metabolomics analysis. The metabolomics analysis revealed that acute-phase tyrosine and phenylalanine were significantly reduced in patients who later developed aSAH-CI, whereas their decarboxylation products—trace amines (such as tyramine and phenylethylamine)—were abnormally elevated. This suggests that the restricted pool of amino acid precursors is potentially shunted toward toxic metabolic branches. First, we hypothesize that the deficiency of precursor substances might restrict cognitive reserve. Tyrosine and phenylalanine are essential precursors for the synthesis of catecholamine neurotransmitters, such as dopamine and norepinephrine. Furthermore, they can influence the rapid activation of catecholaminergic neurons, which has been proven beneficial in the treatment of Parkinson’s disease and depression (Conlay and Zeisel, 1982). During the recovery phase of aSAH, the remodeling of damaged neural circuits urgently requires a substantial supply of neurotransmitters. The reduction of these gut-derived aromatic amino acids may reflect a depletion of the resources necessary for brain repair, which may potentially restrict the recovery of executive function and memory. Furthermore, catecholamines such as dopamine have been shown to improve cognitive impairment (Bonet Olivares et al., 2025). Secondly, the accumulation of trace amines, which are amino acid decarboxylation products, is hypothesized to exacerbate neurological damage. Correlation analysis revealed that the elevation of trace amines was significantly positively correlated with the enrichment of *Enterococcus* B *durans*. Studies have demonstrated that bacteria of the genus *Enterococcus* commonly carry the tyrosine decarboxylase (*tyrDC*) gene, enabling them to efficiently convert tyrosine in the gut into tyramine (Li et al., 2024). This altered metabolic profile is characterized by a concurrent reduction in putatively beneficial tyrosine and an accumulation of tyramine, a potentially neurotoxic metabolite. Although minute quantities of trace amines can function as neuromodulators, excessive accumulation of tyramine and phenylethylamine has been reported to possess sympathomimetic toxicity, often inducing peripheral vasoconstriction and blood pressure fluctuations (Broadley, 2010). Based on these established pharmacological properties, we hypothesize that the aberrant systemic accumulation of gut-derived trace amines may contribute to cerebrovascular instability. Specifically, the sympathomimetic effects and hemodynamic stress exerted by these amines might increase susceptibility to, or exacerbate, cerebral vasospasm following aSAH (Takemoto et al., 2020). In neurosurgical practice, such microvascular spasm is widely recognized as a pivotal factor in the development of delayed cerebral ischemia (DCI) (Macdonald, 2014; Takemoto et al., 2020). We postulate that the superimposition of this putative trace amine-associated secondary ischemia upon the primary brain injury might be closely associated with the exacerbation of cognitive impairment. However, as our current findings reflect associative relationships, further mechanistic investigations are required to definitively establish causality in this pathway.

During the pathophysiological process of aSAH, the toxic effects of subarachnoid blood and the primary brain injury rapidly led to structural disruption and increased permeability of the BBB. Germanó et al. (1992) previously demonstrated using the [14C]-α-aminoisobutyric acid technique that BBB permeability is significantly elevated following aSAH, specifically reaching 1.3–1.5 times that of the control group in the cortical and cerebellar gray matter regions. The impairment of this barrier function opens a “window of danger” for abnormal gut metabolites to potentially exert central neurotoxicity. Under physiological conditions, minute quantities of trace amines are strictly regulated by systems such as monoamine oxidase (Shih et al., 1999). However, under the dual context of physical BBB disruption triggered by aSAH and the BBB attenuation potentially induced by the aforementioned microecological dysbiosis, we speculate that excessively generated trace amines might have the potential to leak into the brain at pathological concentrations. Notably, trace amine-associated receptors (TAARs, particularly TAAR1) are widely expressed in the brain, predominantly within the limbic system, including the hippocampus and amygdala (Rutigliano et al., 2017). At pathological concentrations, tyramine and phenylethylamine have been shown to not only interfere with the normal signal transduction of TAARs on hippocampal neurons within the brain but also directly induce mitochondrial dysfunction and a burst of reactive oxygen species (ROS) in neurons through the activation of these receptors (Tobolski et al., 2022).

Serological data indicated that patients who developed aSAH-CI exhibited a heightened acute state of systemic inflammation, oxidative stress, and apoptosis during the initial 24–48 h post-admission. The abnormally elevated trace amines (such as tyramine) were significantly positively correlated with serum apoptotic factors (BAX and Cyt-C) and significantly negatively correlated with an antioxidant (SOD). While our study cannot assess direct receptor activation within the central nervous system, this correlation aligns with the activation mechanism of TAARs, as excessive intracellular tyramine has been reported to induce mitochondrial dysfunction and trigger a burst of ROS (Tobolski et al., 2022). Although the enrichment of the core pro-inflammatory microbiota *E. lenta* identified in our study exhibited no correlation with serum factors, the depletion of *A. rectalis* and species of the genus *Roseburia* was significantly positively correlated with the reduction in an anti-inflammatory cytokine (IL-10) and antioxidants (SOD and GSH). This suggests that the deficiency of these putative anti-inflammatory bacteria may be associated with an attenuated protective state for cognitive function. The cross-omics Spearman correlation network analysis further supported this finding. Topological degree analysis of the network indicated that within the pathogenic module enriched in aSAH-CI, the previously mentioned phenylethylamine, together with a lipid peroxidation marker (MDA), a pro-inflammatory cytokine (IL-18), and a pro-apoptotic protein (BAX), constituted highly connected hub nodes. Notably, we observed a dense network of negative cross-domain correlations between these two functional modules. This suggests an association between the longitudinal development of cognitive impairment following aSAH and a profound acute-phase imbalance in the gut metabolic profile, characterized by the depletion of putative neuroprotective amino acids and the concurrent accumulation of potentially neurotoxic trace amines. Paralleling the observed dysbiosis in the core microbiota, this metabolic imbalance is significantly correlated with elevated systemic inflammatory, oxidative stress, and apoptotic markers, all of which exhibit a collective association with the longitudinal manifestation of central cognitive impairment.

From a clinical translation perspective, the early identification of patients at high risk for cognitive impairment following aSAH has consistently remained a major challenge in neurosurgical clinical practice. Traditional imaging scoring systems (such as the Fisher grade) often fail to accurately assess cognitive impairment at an early stage. Univariate ROC curve analysis and LOOCV demonstrated that the reduction of *A. rectalis* and the elevation of trace amines (such as tyramine) in the feces exhibited excellent diagnostic efficacy. Furthermore, integrating these factors with the host’s systemic inflammatory, oxidative stress, and apoptotic states offers a potential multi-dimensional approach to predicting cognitive impairment in patients with aSAH (e.g., comatose patients) prior to the onset of irreversible neurological deficits. Concurrently, it also lays a solid theoretical foundation for the future development of therapeutic strategies based on targeted interventions of the gut microbiota and its metabolites.

## 5 Conclusion

**FIGURE 8:**
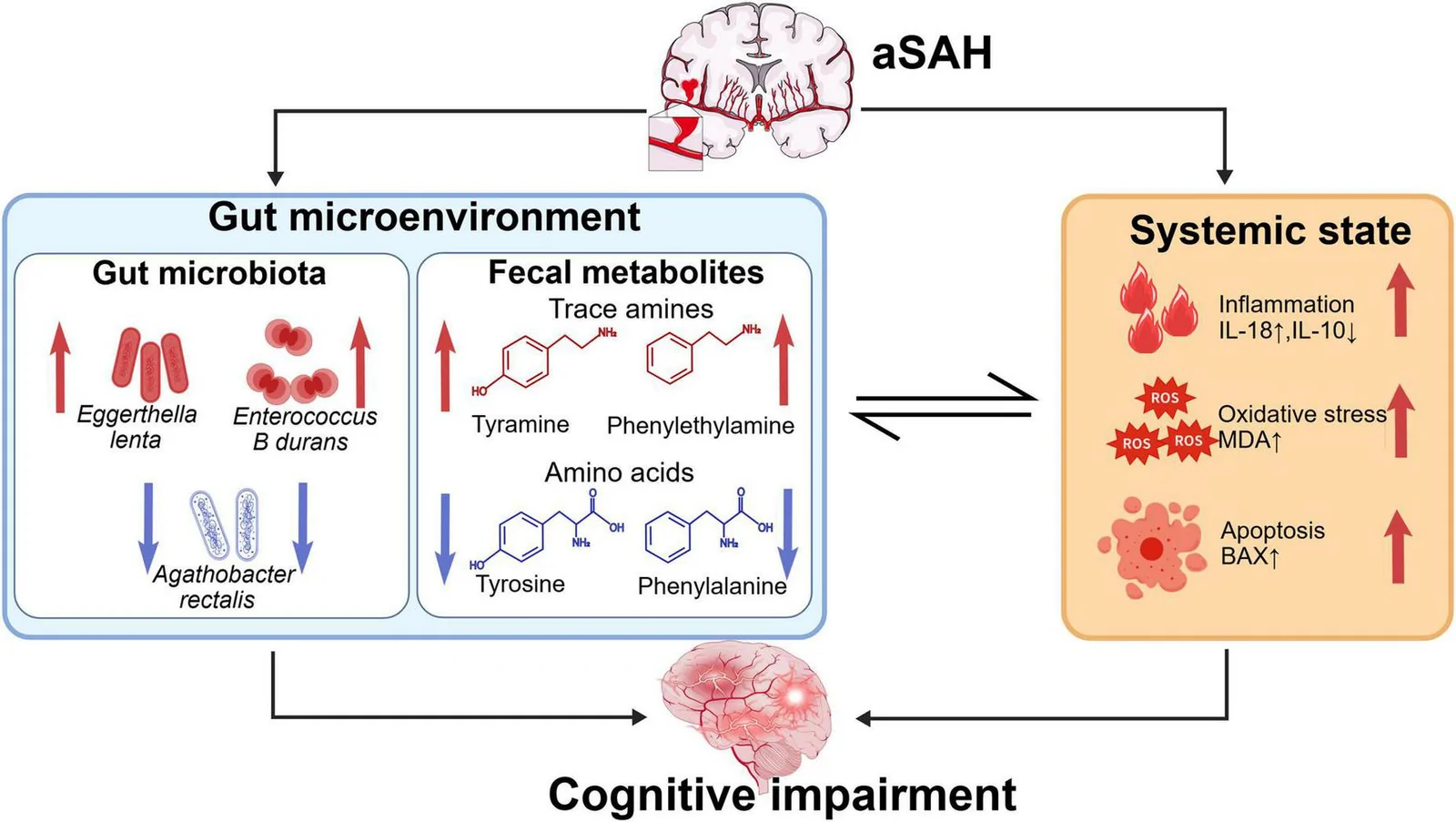
Schematic illustration of the proposed “gut-brain axis double-hit mechanism” associated with cognitive impairment following aSAH. These alterations in gut microbiota and dysregulated metabolites, along with host systemic inflammation, oxidative stress, and neuronal apoptosis, are closely correlated with the chronic cognitive deficits following aSAH. Created with BioGDP.com (Jiang et al., 2025).

In summary, by integrating multi-omics analysis, the present study revealed a significant cross-system association between gut microecological dysbiosis and cognitive impairment following aSAH. Our results indicate that the clinical profile of cognitive impairment following aSAH may exhibit a potential “gut-brain axis double-hit signature.” Based on our data, this is manifested by the significant depletion of putative protective commensal bacteria responsible for amino acid synthesis (*A. rectalis*) and the abnormal expansion of potential pathogenic bacteria possessing amino acid decarboxylation capabilities (*Enterococcus* B *durans*, *E. lenta*). The altered profile of these specific micro-ecological functional modules is closely associated with an imbalance in aromatic amino acid metabolism, characterized by the concurrent enrichment of trace amines with putative neurotoxicity (such as tyramine and phenylethylamine). These abnormally accumulated specific metabolites and the altered gut microenvironment are highly correlated with the host’s systemic inflammatory activation, exacerbated oxidative stress, and neural cell apoptotic cascades (elevated levels of BAX, MDA, and IL-18) (Figure 8).

Furthermore, the multi-omics integrative diagnostic model constructed in this study highlights the substantial clinical potential of specific gut microbial and trace amine signatures as early, non-invasive predictive biomarkers. These findings not only provide profound insights into the systemic pathophysiological mechanisms of the “gut-brain axis” potentially implicated in cognitive decline following aSAH, but also provide a novel translational medical perspective for the future management of neurosurgical critical care, aiming to remodel the metabolic microenvironment through targeted gut decarboxylase inhibitors or precision probiotic interventions, thereby potentially improving the long-term cognitive prognosis of patients with aSAH.

## 6 Limitations

First, regarding cohort representativeness and clinical translation, constrained by the single-center exploratory design, limited sample size, an objective sampling bias (18 missing fecal samples due to acute neurocritical constraints), and potential environmental confounding factors such as the daily nutritional intake, dietary composition, and dietary preferences, the core biomarkers identified in this study currently lack validation in an independent external cohort. Additionally, despite the statistical corrections applied, the combination of a relatively small sample size and high-dimensional multi-omics data carries inherent risks of false positives and statistical overfitting. Therefore, the construction of complex multivariate predictive models and the definitive validation of these biomarkers will be deferred to future multicenter, large-scale cohorts. Second, concerning omics technology and analytical depth, the current conclusions are primarily derived from multi-omics correlation networks. Given the limited sensitivity of untargeted LC-MS for volatile small molecules such as short-chain fatty acids, future studies need to employ targeted gas chromatography-mass spectrometry (GC-MS) for precise quantification (Papadimitropoulos et al., 2018). Furthermore, metatranscriptomics should be introduced to quantify the expression abundance of specific genes (such as *tyrDC*). Finally, regarding the confirmation of causal mechanisms, it is important to acknowledge that the mechanistic pathways proposed in our discussion, such as the specific neurotoxic effects of trace amines on the central nervous system, remain speculative and are inferred from existing literature rather than directly proven by our multi-omics data. Our current findings are restricted to predictive associations rather than concurrent causal relationships, primarily due to the temporal mismatch between acute-phase omics sampling and chronic-phase cognitive assessments. Without direct pathological evidence, it is imperative that future studies employ parallel longitudinal tracking in conjunction with targeted fecal microbiota transplantation (FMT) using germ-free mice, or employ genetic engineering techniques for the colonization of mutant strains with specific decarboxylase knockouts, in order to confirm the causal role of gut-derived trace amines in contributing to the development of cognitive impairment following aSAH.

## Data Availability

The raw metagenomic sequencing data generated in this study can be found in the NCBI Sequence Read Archive (https://www.ncbi.nlm.nih.gov/), accession PRJNA1495175. The untargeted metabolomics data can be found in MetaboLights (https://www.ebi.ac.uk/metabolights/), accession MTBLS15087.
