# Supplementary material for: Gut microbiota dysbiosis and aromatic amino acid metabolism alterations: a multi-omics analysis of cognitive impairment following aneurysmal subarachnoid hemorrhage
Source: Front Microbiol. 2026 Aug 18;17:1870309. doi: 10.3389/fmicb.2026.1870309 (PMC13529724; doi:10.3389/fmicb.2026.1870309)
Supplement: Supplementary file 1 [file Data_Sheet_1.docx]

Supplementary Materials

# 1 **Supplementary Method 1**

Inclusion criteria for aneurysmal subarachnoid hemorrhage (aSAH) patients: (1) a confirmed diagnosis of intracranial aneurysmal rupture and hemorrhage based on head computed tomography (CT), computed tomography angiography (CTA), or digital subtraction angiography (DSA); (2) age between 18 and 65 years; (3) a Hunt-Hess grade of I–III (mild to moderate) at admission; (4) receipt of interventional treatment without developing severe complications postoperatively (e.g., postoperative coma or the need for a repeat surgical intervention); (5) completion of at least an elementary school education (to ensure the validity of subsequent cognitive or functional scale assessments); (6) availability of complete baseline clinical, laboratory, and imaging data.

Patients with unruptured intracranial aneurysm (UIA) were recruited concurrently to serve as the control group. The inclusion criteria for this group were: (1) a definitive diagnosis of an unruptured intracranial aneurysm based on head CT, CTA, or DSA; (2) aged between 18 and 65; (3) receipt of an interventional procedure without experiencing severe postoperative complications (e.g., postoperative coma or the need for a repeat surgical intervention); (4) availability of complete baseline clinical, laboratory, and imaging data; (5) strict matching with the aSAH group in terms of the distribution of factors such as age, sex, educational level, comorbidities, and aneurysm characteristics (*P* > 0.05).

Because gut microbiota and cognitive assessments are highly sensitive to clinical confounding factors, participants in either group were excluded if they met any of the following criteria: (1) microbiome-altering factors: systemic administration of broad-spectrum antibiotics, probiotics, or prebiotic preparations within 3 months prior to admission; any clinical antibiotic exposure (including prophylactic or empirical administration for suspected infections or fever workups) from the time of admission to the exact moment of fecal sample collection (24-48 hours); coexisting chronic gastrointestinal diseases (e.g., inflammatory bowel disease); recent gastrointestinal surgery; or extreme dietary habits (e.g., long-term strict vegetarianism, or explicitly poor diets characterized by severely low fiber intake); or prolonged fasting or nil per os (NPO) status prior to admission; (2) neurological and psychiatric disorders: a history or family history of stroke or symptomatic cerebrovascular disease; a confirmed diagnosis of neurodegenerative diseases (e.g., Alzheimer’s or Parkinson’s disease), major psychiatric disorders (e.g., severe depression or schizophrenia), or a history of cognitive impairment; or concomitant severe organic intracranial lesions (e.g., brain tumors, severe traumatic brain injury, or hydrocephalus); (3) major systemic diseases: underlying conditions such as severe cardiac, hepatic, or renal failure; (4) impairments affecting scale-based assessments: severe aphasia, severe visual or auditory impairments, or other conditions preventing cooperation with neuropsychological assessments; (5) incomplete follow-up data or loss to follow-up.

# 2 **Supplementary Method 2**

2.1 **Metagenomic sequencing**

Total microbial genomic DNA was extracted using the MagBeads FastDNA Kit for Soil (MP Biomedicals, Irvine, CA, USA) and stored at −20°C. DNA quantity and quality were assessed using a Qubit 4 Fluorometer (Invitrogen, Carlsbad, CA, USA) and agarose gel electrophoresis. Metagenomic shotgun sequencing libraries (insert sizes: 400 bp) were constructed using the Illumina TruSeq Nano DNA LT Library Preparation Kit and sequenced on a DNBSEQ-T7 platform using a PE150 strategy (Personal Biotechnology Co., Ltd., Shanghai, China). Raw reads were processed to generate quality-filtered reads using fastp (-1 50 -g -W 5 -3 -5 -q 20 -u 30). Subsequently, the reads were aligned to the human reference genome utilizing minimap2 (-ax sr) to remove host-derived contamination. Taxonomic classification was performed using Kraken2 (--confidence 0.5) against a customized GTDB database. For de novo assembly, MEGAHIT (v1.1.2) was employed (--k-list 33,55,77,99,127 --min-contig-len 300); reads that failed to align to the assembled contigs were pooled and reassembled. Gene prediction was conducted using Prodigal (-p meta). Predicted protein sequences were clustered using MMseqs2 (easy-linclust mode: -c 0.9 --cov-mode 1 --min-seq-id 0.95 --cluster-mode 2). The lowest common ancestor (LCA) taxonomy of non-redundant genes was determined using MMseqs2 (taxonomy mode: --lca-mode 3 -s 2) against the customized GTDB database. To assess gene abundances, high-quality reads were mapped onto contigs using minimap2 (-ax sr), and read counts were computed using HTSeq, followed by normalization to counts per million (CPM). Functional annotation was accomplished through MMseqs2 (search mode: -s 5.7) against the Kyoto Encyclopedia of Genes and Genomes (KEGG) database.

2.2 **Non-targeted metabolomics sequencing**

Metabolites were extracted from 20 mg of each sample using 300 µL of pre-cooled methanol (containing 5 ppm 2-chlorophenylalanine). The mixture was homogenized (55 Hz, 60 s, twice), sonicated for 10 min, and incubated at −20°C for 30 min. Following centrifugation (12,000 rpm, 4°C, 10 min), the supernatants were filtered through a 0.22-µm membrane. LC-MS/MS analysis was performed using an ACQUITY UPLC HSS T3 column (1.8 µm, 2.1 × 100 mm) at 40°C with an 8.5-min elution gradient consisting of 0.1% formic acid in water and acetonitrile. Data acquisition was conducted on a Thermo Orbitrap Exploris 120 mass spectrometer in both positive and negative heated electrospray ionization (HESI) modes (3.5 kV/−3.0 kV, capillary temperature 320°C). Full MS scans were acquired at a resolution of 60,000 (80–1000 m/z), followed by top-four data-dependent acquisition (DDA) MS/MS (resolution: 15,000; higher-energy collisional dissociation [HCD]: 30%; dynamic exclusion: 4 s). Pooled quality control (QC) samples were injected every 6 to 12 samples to monitor system stability. Raw data were processed using MS-DIAL (v4.9.221218) for peak extraction, alignment, gap-filling, and normalization. Metabolites were identified against the PSNGM, mzCloud, LIPID MAPS, HMDB, MoNA, NIST_2020_MSMS, and AI-predicted spectral libraries using parameters including an MS1 tolerance of 0.01 Da, an MS2 tolerance of 0.05 Da, a minimum peak height of 10,000, and an identification score cut-off of 70.

2.3 **Serum biomarker testing**

The serum concentrations of inflammatory factors (interferon-gamma [IFN-γ], interleukin-1 beta [IL-1β], interleukin-6 [IL-6], interleukin-10 [IL-10], interleukin-18 [IL-18], and tumor necrosis factor-alpha [TNF-α]) and apoptosis indicators (Bcl-2-associated X protein [BAX], caspase-3, and cytochrome c [Cyt-C]) were determined using specific ELISA kits. Briefly, calibration standards were assayed concurrently with the samples to generate a standard curve. Standards and testing samples were added to the wells, covered with an adhesive strip, and incubated for 30 minutes at 37°C. After aspirating and washing each well five times, the horseradish peroxidase (HRP)-conjugate reagent was added and incubated for 30 minutes at 37°C. Following another five washes, chromogen solutions A and B were added and incubated for 15 minutes at 37°C. The reaction was terminated by the addition of a stop solution. The optical density (OD) was measured at 450 nm using a microtiter plate reader within 15 minutes. The concentrations of the markers in the samples were then determined by comparing the OD of the samples to the standard curve. Additionally, serum oxidative stress indicators, including malondialdehyde (MDA), superoxide dismutase (SOD), and glutathione (GSH), were quantified using corresponding microplate assay kits according to the manufacturer's protocols. Briefly, MDA levels were determined using the thiobarbituric acid (TBA) method, calculated by the difference in absorbance at 532 nm and 600 nm. SOD activity was evaluated utilizing the WST-8 method, which measures the inhibition of water-soluble formazan dye formation at 450 nm. GSH concentrations were assessed based on their specific reaction with 5,5′-dithiobis-(2-nitrobenzoic acid) (DTNB), with the absorbance of the resulting complex measured at 412 nm.

# 3 **Supplementary Method 3**

3.1 **Post-hoc power analysis**

To validate the adequacy of our sample size, a post-hoc power analysis was conducted based on the key multi-omics markers among the three study cohorts. Prior to analysis, the relative abundances of the microbiome and metabolome data underwent log_2_(x + 1) transformation to satisfy normality assumptions. For the core gut microbiota (*Agathobacter rectalis*), one-way analysis of variance (ANOVA) revealed an exceptionally robust effect size (Cohen's *f* = 1.08). Based on a *α* level of 0.05 and a desired statistical power of 90%, the power analysis indicated a minimum total sample size of 15 (5 per group) to detect significant inter-group heterogeneity. Similarly, for the core metabolite (tyramine), the calculated effect size reached a large level (Cohen's *f* = 0.78), necessitating a minimum total sample size of 24 (8 per group) to achieve 90% power. Consequently, our current multi-omics cohort comprising 30 patients (11 vs. 10 vs. 9) substantially exceeds the requisite threshold, ensuring robust statistical power (1- *β* > 0.90) to capture the significant differences in these primary endpoints.

3.2 **Confounding factor adjustment via Analysis of Covariance (ANCOVA)**

Given the significant baseline imbalances in the prevalence of hypertension and clinical complications among the study groups, an ANCOVA was strictly implemented to evaluate the inter-group differences and eliminate potential confounding effects. Prior to the adjustment, the raw concentration data of serum biomarkers were subjected to a log_2_(x + 1) transformation to satisfy the assumptions of normality and homogeneity of variance. The ANCOVA model utilized the transformed data as the dependent variable, 'Group' as the fixed factor, and 'Hypertension' and 'Complications' as categorical covariates. To account for the unbalanced sample sizes across groups, the Type III sum of squares was applied. Post-hoc pairwise comparisons were subsequently conducted based on Estimated Marginal Means (EMMs). To rigorously account for multiple testing, all resulting *P*-values were corrected using the False Discovery Rate (FDR), with an adjusted *P* < 0.05 considered statistically significant.

3.3 **Multivariable sensitivity analysis for multi-omics biomarkers**

Given the selection bias (age, diabetes, and aneurysm location) between the included (n = 30) and excluded (n = 18) cohorts, a multivariable sensitivity analysis was rigorously executed to test the robustness of the core correlative findings. Prior to analysis, the relative abundance of *Agathobacter rectalis* and the quantitative levels of tyramine underwent a log_2_(x + 1) transformation to satisfy the assumption of normality. An ANCOVA model was constructed utilizing the transformed multi-omics data as the dependent variables and the 'Cognitive Outcome' as the fixed factor. Crucially, 'Age', 'Diabetes', and 'Aneurysm location' were simultaneously incorporated as covariates to strictly control for their confounding effects driven by the sampling bias. The Type III sum of squares was applied to account for the unbalanced design. Subsequent post-hoc pairwise comparisons were conducted based on EMMs, and all resulting *P*-values were adjusted using the FDR to strictly control the false positive rate. Specifically, differences in *Agathobacter rectalis* (Adjusted overall *P* < 0.001) and tyramine (Adjusted overall *P* = 0.009) were robustly preserved. And these findings confirm that the aSAH-CI group exhibits independently and significantly distinct levels of these core biomarkers compared to the aSAH-WCI group (*P* < 0.05).

# 4 **Supplementary Method 4**

4.1 **Metagenomic Diversity and Taxonomic Profiling**

Microbial species diversity was assessed using the vegan and ggplot2 packages in R (v4.5.2). To validate the sequencing depth, rarefaction curves (Shannon diversity index curves) were generated by randomly sampling species-level abundance distributions at different sequencing depths. Richness was quantified using the Chao1 index; diversity was evaluated using the Shannon and Simpson indices; and evenness was measured using Pielou’s evenness index. Statistical differences in alpha-diversity (α-diversity) among the three groups were assessed using the nonparametric Kruskal-Wallis rank-sum test, followed by Dunn’s *post hoc* test and FDR correction for multiple comparisons. For beta-diversity (β-diversity), principal coordinate analysis (PCoA) was performed based on Bray-Curtis distance matrices to quantify differences in community composition and spatial distribution patterns, and the results were visualized using R and QIIME software. The significance of spatial separation was rigorously tested using permutational multivariate analysis of variance (PERMANOVA, 999 permutations). For pairwise comparisons between multiple groups, the resulting *P*-values were adjusted using the Benjamini-Hochberg (BH) FDR method. Additionally, the VennDiagram package in R was utilized to identify and visualize the number of shared and unique species across groups.

4.2 **Differential Cluster Identification and LEfSe Analysis**

To identify specific microorganisms driving differences between groups, taxonomic abundance profiles were first normalized. Differentially abundant taxa were identified using the Kruskal-Wallis test or Student’s *t*-test, and the BH method was applied to control the FDR. Taxa with an adjusted *P*-value < 0.05 were considered significantly different. Subsequently, biomarker discovery was performed using the linear discriminant analysis effect size (LEfSe) package in Python and the ggtree package in R. Briefly, the Kruskal-Wallis test was used to identify taxa with significant differential abundance across groups. To effectively control the FDR inherent in high-dimensional omics data, the resulting P-values were adjusted using the BH method. This was followed by the Wilcoxon rank-sum test to assess the significance of pairwise between-group differences. Linear discriminant analysis (LDA) was then conducted on the identified differentially abundant taxa to estimate their effect size on intergroup differences. An LDA score threshold of > 2.0 and a *P*-value < 0.05 were established to define key biomarker taxa. The phylogenetic relationships and hierarchical distribution of these key biomarkers were visualized using a LEfSe cladogram, and species-level biomarkers were individually presented using LDA bar charts.

4.3 **Metagenomic Functional Profiling and Species-Function Contribution Analysis**

The functional potential of microbial communities was decoded using the KEGG database. High-quality sequencing reads were aligned to a non-redundant gene catalog, and pathway abundances at the L2 and L3 levels were calculated and normalized to transcripts per million (TPM). Differences in functional abundance were analyzed using the Kruskal-Wallis test or Student’s *t*-test, with the FDR controlled using the BH method (adj. *P* < 0.05). Association analyses between species and functions were conducted based on the relative abundances of species and their corresponding functional annotations to determine species-specific contributions. This approach decomposed the relative abundance of specific KEGG pathways and mapped them back to specific microbial contributors at the phylum and species levels, thereby precisely quantifying the biological impact of key microbial taxa on the host’s metabolic reservoir.

4.4 **Non-targeted metabolomics and pathway enrichment analysis**

Multivariate statistical analysis of metabolomics data was performed using the ropls package in R (v4.5.2). Supervised partial least squares discriminant analysis (PLS-DA) was employed to address high-dimensional multicollinearity. To rigorously validate the model’s robustness and mitigate the risk of overfitting, 200 permutation tests were conducted, with a comprehensive evaluation based on two core metrics: cumulative explanatory power (*R*²) and predictive power (*Q*²). Core differential metabolites were identified based on a dual-filter criterion: variable importance in projection (VIP) scores > 1.0 and *P* < 0.05, adjusted using the BH FDR method. Venn diagrams were used to illustrate the overlap among differentially abundant metabolites across groups. Subsequently, KEGG pathway enrichment analysis was performed. Fisher’s exact test was used to calculate the significance of metabolite enrichment in specific pathways, and the top 20 significant metabolic pathways with the lowest BH-adjusted *P*-values (FDR < 0.05) were selected for visualization.

4.5 **Multi-omics Association Networks and Evaluation of Diagnostic Performance**

To elucidate the interactions among gut microbiota, fecal metabolites, and serum pathophysiological biomarkers, Spearman’s rank correlation coefficients were calculated for all pairs of variables. To rigorously control for the FDR inherent in massive cross-omics comparisons, the resulting *P*-values were adjusted using the BH method. Strict selection criteria (*P* < 0.05 and |*r*| > 0.5) were applied, and the resulting correlation matrix was visualized using an association heatmap (with significance levels of *P* < 0.05, *P* < 0.01, and *P* < 0.001 clearly labeled). Based on the above results, a cross-omics association network was constructed. Network topological properties (primarily degree centrality) were calculated using the igraph package in R (v4.5.2). The network was visualized using Gephi, with node size proportional to degree centrality, intuitively highlighting “hub nodes” with high connectivity. Edge colors indicated positive or negative statistical correlations. Finally, to evaluate the clinical diagnostic value of the identified key biomarkers (such as *Agathobacter rectalis*, tyramine, BAX, MDA, and IL-10), univariate receiver operating characteristic (ROC) curve analysis and leave-one-out cross-validation (LOOCV) were performed using the pROC package in R. The diagnostic performance of each biomarker in the early prediction of aSAH-induced high inflammatory states was comprehensively quantified by calculating the area under the curve (AUC) and its 95% confidence interval (95% CI).

# 5 **Supplementary Table S1**

**Table S1. Baseline clinical and demographic characteristics of patients with and without available fecal samples**

| Characteristics | Available fecal samples | Missing fecal samples | *P* value |
| --- | --- | --- | --- |
| Sex, n (%) |  |  | 0.493 |
| Male | 13 (43.3%) | 6 (33.3%) |  |
| Female | 17 (56.7%) | 12 (66.7%) |  |
| Age, mean ± SD | 56.97 ± 4.51 | 50.00 ± 6.25 | 0.001 |
| BMI, n (%) |  |  | 0.705 |
| Underweight | 1 (3.3%) | 0 (0.0%) |  |
| Healthy weight | 13 (43.3%) | 7 (38.9%) |  |
| Overweight | 14 (46.7%) | 8 (44.4%) |  |
| Obesity | 2 (6.7%) | 3 (16.7%) |  |
| Education, n (%) |  |  | 0.113 |
| ≤ Primary | 11 (36.7%) | 5 (27.8%) |  |
| Junior high | 8 (26.7%) | 11 (61.1%) |  |
| Senior high | 5 (16.7%) | 1 (5.6%) |  |
| ≥ College | 6 (20.0%) | 1 (5.6%) |  |
| Smoking, n (%) |  |  | 0.376 |
| No | 22 (73.3%) | 11 (61.1%) |  |
| Yes | 8 (26.7%) | 7 (38.9%) |  |
| Drinking alcohol, n (%) |  |  | 0.468 |
| No | 25 (83.3%) | 13 (72.2%) |  |
| Yes | 5 (16.7%) | 5 (27.8%) |  |
| Hypertension, n (%) |  |  | 0.697 |
| No | 10 (33.3%) | 7 (38.9%) |  |
| Yes | 20 (66.7%) | 11 (61.1%) |  |
| Diabetes, n (%) |  |  | 0.017 |
| No | 18 (60.0%) | 17 (94.4%) |  |
| Yes | 12 (40.0%) | 1 (5.6%) |  |
| Dyslipidemia, n (%) |  |  | 1 |
| No | 29 (96.7%) | 18 (100.0%) |  |
| Yes | 1 (3.3%) | 0 (0.0%) |  |
| Hunt-hess, n (%) |  |  | 0.076 |
| Ⅰ | 6 (28.6%) | 1 (8.3%) |  |
| Ⅱ | 10 (47.6%) | 3 (25.0%) |  |
| Ⅲ | 5 (23.8%) | 8 (66.7%) |  |
| Fisher, n (%) |  |  | 0.542 |
| 1 | 4 (19.0%) | 1 (8.3%) |  |
| 2 | 13 (61.9%) | 6 (50.0%) |  |
| 3 | 1 (4.8%) | 1 (8.3%) |  |
| 4 | 3 (14.3%) | 4 (33.3%) |  |
| Complications, n (%) |  |  | 0.071 |
| No | 6 (20.0%) | 8 (44.4%) |  |
| Yes | 24 (80.0%) | 10 (55.6%) |  |
| Aneurysm |  |  |  |
| Size, mean ± SD | 7.51 ± 2.76 | 8.95 ± 2.84 | 0.091 |
| Height-width ratio, mean ± SD | 1.67 ± 0.53 | 1.67 ± 0.24 | 0.979 |
| Location, n (%) |  |  | 0.036 |
| Anterior circulation | 23 (76.7%) | 18 (100.0%) |  |
| Posterior circulation | 7 (23.3%) | 0 (0.0%) |  |
| Arterial bifurcation, n (%) |  |  | 0.388 |
| No | 25 (83.3%) | 17 (94.4%) |  |
| Yes | 5 (16.7%) | 1 (5.6%) |  |
| Multiple aneurysms, n (%) |  |  | 0.724 |
| No | 24 (80.0%) | 13 (72.2%) |  |
| Yes | 6 (20.0%) | 5 (27.8%) |  |
| PPIs, n (%) |  |  | 0.551 |
| No | 14 (46.7%) | 10 (55.6%) |  |
| Yes | 16 (53.3%) | 8 (44.4%) |  |
| Opioids, n (%) |  |  | 0.878 |
| No | 19 (63.3%) | 11 (61.1%) |  |
| Yes | 11 (36.7%) | 7 (38.9%) |  |
| Laxatives, n (%) |  |  | 0.599 |
| No | 16 (53.3%) | 11 (61.1%) |  |
| Yes | 14 (46.7%) | 7 (38.9%) |  |

# 6 **Supplementary Figure S1**

**Figure S1. Functional profiling of the gut metagenomes and overall clustering of fecal metabolites among the three patient cohorts.** (A) Bar plots showing the relative abundance of differential functional pathways at KEGG level 1. (B) Bar plots showing the relative abundance of differential functional pathways at KEGG level 2. (C) Bar plots showing the relative abundance of differential functional pathways at KEGG level 3. (D) Overall metabolite clustering heatmap in positive ion mode (POS). (E) Overall metabolite clustering heatmap in negative ion mode (NEG). (F) Validation of the partial least squares-discriminant analysis (PLS-DA) model.
